# NICD3 regulates the expression of *MUC5AC* and *MUC2* by recruiting SMARCA4 and is involved in the differentiation of mucinous colorectal adenocarcinoma

**DOI:** 10.1002/1878-0261.13296

**Published:** 2022-08-11

**Authors:** Xiaodong Yan, Yuan Cheng, Xia Zhang, Yi Hu, Haixia Huang, Jie Ren, Boye Wen, Yuhui Yang, Keyuan Xiao, Wenqing Hu, Wei Wang

**Affiliations:** ^1^ Department of Physiology and Pathophysiology, School of Basic Medical Sciences Capital Medical University Beijing China; ^2^ Department of Gastrointestinal Surgery Changzhi People's Hospital, The Affiliated Hospital of Shanxi Medical University Changzhi China; ^3^ Fuxing Hospital Capital Medical University Beijing China; ^4^ Department of Immunology, School of Basic Medical Sciences Capital Medical University Beijing China; ^5^ School of Basic Medical Sciences Capital Medical University Beijing China; ^6^ Central Laboratory Changzhi People's Hospital, The Affiliated Hospital of Shanxi Medical University Changzhi China; ^7^ Beijing Lab for Cardiovascular Precision Medicine Capital Medical University Beijing China

**Keywords:** MUC2, MUC5AC, mucinous colorectal adenocarcinoma, NOTCH3, SMARCA4

## Abstract

Adenocarcinoma is the most prevalent histological subtype of colorectal cancer (CRC), with mucinous colorectal adenocarcinoma (MCA) being a unique form. Although the mucinous subtype is known to elicit a worse response to chemotherapy and immunotherapy than the nonmucinous subtype, its pathogenesis remains poorly understood. Neurogenic locus notch homolog protein 3 (NOTCH3), a member of the NOTCH subfamilies, is highly expressed in CRC. In the past three decades, many studies have been performed evaluating the biological role of NOTCH3 in CRC. However, the precise activities of NOTCH3 in MCA, as well as the mechanisms involved in its transcriptional control, are yet to be elucidated. Our finding showed that the critical transcriptional regulatory factor transcription activator BRG1 (SMARCA4) directly binds to the intracellular domain of NOTCH3 to control transcriptional regulation. Moreover, RNA‐sequencing results indicated a common targeting effect on the transcriptional activity of mucin‐5AC (*MUC5AC*) and mucin‐2 (*MUC2*) in CRC cells by NOTCH3 and SMARCA4. Furthermore, NOTCH3 was found to control the expressions of *MUC5AC* and *MUC2* in a SMARCA4‐dependent manner. *MUC5AC* and *MUC2*, which encode two secreted mucins, are located on chromosome 11p15.5, and are linked to the development of MCA. This finding suggests that the interaction between NOTCH3 and SMARCA4 may be involved in MCA differentiation by jointly targeting *MUC5AC* and *MUC2*. Patients with MCA are often treated in accordance with CRC guidelines. Determining the relationship between NOTCH3 and SMARCA4 by demonstrating their interactions in the pathophysiology of MCA could provide novel therapeutic targets and help identify potential prognostic markers for MCA.

## Graphical abstract

Neurogenic locus notch homolog protein 3 (NOTCH3) is highly expressed in colorectal cancer (CRC). NOTCH3 may target *MUC5AC* and *MUC2* by recruiting transcription activator BRG1 (SMARCA4) to regulate the growth, invasion, and migration of CRC cells, which involving in the differentiation and development of mucinous colorectal adenocarcinoma (MCA), indicating a novel molecular mechanism of occurrence of MCA mediating by NOTCH3.

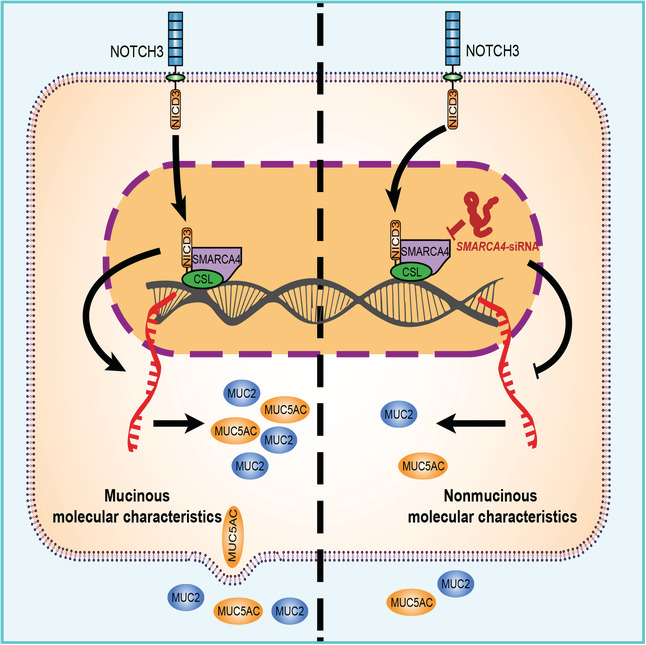

**Abbreviations**
co‐IP: coimmunoprecipitation
CRC: colorectal cancer
DEG: differentially expressed gene
EMT: epithelial–mesenchymal transition
FDR: false discovery rate
GSEA: gene set enrichment analysis
GST: glutathione *S*‐transferase
IHC: immunohistochemistry
MCA: mucinous colorectal adenocarcinoma
PMSF: phenylmethane‐sulfonyl fluoride
PVDF: polyvinylidene fluoride
qRT‐PCR: quantitative real‐time PCR
RNA‐seq: RNA deep‐sequencing
siRNA: small‐interfering RNA

## 1 Introduction

Colorectal cancer (CRC) is the fourth most deadly disease in the world, and kills approximately 900 000 people annually. In addition to the aging population and the deterioration of people's dietary habits, obesity, smoking, and lack of physical exercise exacerbate the risk for CRC [1]. CRC pathogenesis is influenced by both environmental and genetic factors. Therefore, the exploration of specific molecular mechanisms associated with the occurrence and development of CRC is of great significance for developing a more reasonable individualized treatment plan for patients as well as for accelerating the rate of clinical diagnosis and prognosis to improve the survival rate of patients.

In recent decades, CRC treatment has become more individualized owing to the advancement in genomics and molecular pathology of cancer biomarkers, which warrants the refinement of the cancer subtype classification according to its histological and genetic characteristics for strategizing an effective treatment plan. Adenocarcinoma is the most prevalent histological subtype of CRC, with mucinous adenocarcinoma being a unique subtype defined by a significant number of mucinous components accounting for more than 50% of the tumor size [2]. Statistical results suggest that the mucinous adenocarcinoma subtype accounts for 10–20% of all CRC cases [3]. The mucinous subtype affects a larger proportion of women and young CRC patients when compared to the nonmucinous subtype [4, 5, 6]. In addition, mucinous colorectal adenocarcinoma (MCA) is already advanced at the time of diagnosis and usually responds poorly to chemotherapy and immunotherapy [3, 7, 8]. Therefore, the study of the molecular mechanism behind the occurrence and development of MCA is expected to provide a more accurate target for the efficient diagnosis and treatment of MCA.

The excessive activation of the NOTCH pathway is involved in the high occurrence and development of CRC. The pathway is implicated in the regulation of cell‐to‐cell communication, and the dynamic control of  eukaryotic proliferation, differentiation, and apoptosis of the cell in various tissues, especially in CRC tissues and cells [9, 10, 11, 12, 13, 14, 15]. NOTCH family proteins share conserved and similar structures, including the N‐terminal extracellular domain (ECD), transmembrane domain (TMD), and C‐terminal NOTCH intracellular domain (NICD) [16]. When activated, the NOTCH receptor is converted the activated form of NICD and is released into the cytoplasm and further into the nucleus. Subsequently, the receptor forms a transcriptional activator with CSL/RBPJκ (CSL), a transcriptional repressor converted by NOTCH into an activator, which in turn activates the target gene and other transcription factors [17]. Four isoforms of NOTCH receptors are present in mammals, namely, NOTCH1, NOTCH2, NOTCH3, and NOTCH4 [18]. The NOTCH3 receptor was identified for the first time in the neuroepithelium [19]. This receptor is significantly expressed in several malignancies, including CRC, gastric cancer, and pancreatic cancer, according to a growing number of studies [20]. Moreover, the high expression of NOTCH3 has been associated with an increased growth rate of tumors, and the knockdown of NOTCH3 has been reported to significantly reduce the growth rate of CRC [21]. Furthermore, NOTCH3 can induce the migration and invasion of CRC cells by upregulating the exchange factor ASEF [22]. However, the specific functions of NOTCH3 in MCA remain largely unknown.

Previous research has confirmed that the activation of the NOTCH pathway necessitates the involvement of many transcription‐regulating factors [23]. Moreover, the chromatin structure is important for the activity of transcription factors [24]. The transcription activator BRG1, namely SWI/SNF‐related, matrix‐associated, actin‐dependent regulator of chromatin, subfamily a, member 4 (SMARCA4), is a core subunit of the SWI/SNF chromatin‐remodeling complex that regulates transcription via the remodeling of chromatin structures and is involved in immune response, inflammation, and embryonic development [25, 26, 27]. SMARCA4 plays distinct roles in different tissues, and its transcriptional role in binding to the chromatin can be regulated based on structural and functional alterations [28, 29, 30, 31]. Moreover, SMARCA4 promotes the progression of CRC, and a high level of SMARCA4 is linked to a poor prognosis in patients CRC [32]. Additionally, studies have also demonstrated that SMARCA4 is involved in the regulation of the NOTCH1 signaling pathway [23, 33, 34]. However, whether the activation of NOTCH3 requires SMARCA4 in CRC is yet to be elucidated.

In the present study, a direct interaction between NOTCH3 intracellular domain (NICD3) and SMARCA4 in CRC was noted, which indicates that NOTCH3 requires the participation of SMARCA4 in transcriptional regulation. Furthermore, according to RNA‐seq results, NOTCH3 and SMARCA4 displayed a shared targeting impact on the transcriptional activation of mucin 5AC (*MUC5AC*) and mucin 2 (*MUC2*) in CRC cells. The secretory mucin coding genes *MUC5AC* and *MUC2* have been linked to the presence of MCA on chromosome 11p15.5 [35]. Significant differences have been reported in the molecular profiles of MCA and non‐MCA, which indicates their distinct carcinogenic mechanisms. The increased expression of MUC5AC and MUC2 is one of the most obvious molecular features that distinguish MCA from non‐MCA [3, 36, 37, 38, 39]. Our results showed that the interaction between NOTCH3 and SMARCA4 can impact the expressions of MUC5AC and MUC2, which may be critical for MCA differentiation. Patients with MCA are treated as per the same standard treatment guidelines as CRC, and poor responses to current chemotherapy and immunotherapy are often reported. Therefore, specialized and individualized treatment is the need of the hour. Exploring new targets for refining the CRC subtypes is undeniably of great significance for improving the clinical prognosis and therapeutic outcomes in patients with MCA.

## 2 Materials and methods

### 2.1 Human colorectal specimens

The CRC and adjacent tissues (112 patients) were acquired from the Changzhi People's Hospital, The Affiliated Hospital of Changzhi Medical College during 2015–2020. Immediately after collection, all samples were stored in an ultra‐low‐temperature refrigerator at −80 °C. The study was conducted according to the Good Clinical Practice guidelines and in adherence to the Declaration of Helsinki. All patients provided informed consent for a sampling of their tissues. The formalin‐fixed paraffin‐embedded colorectal resection specimens were transferred to a new stone wax block to reconstruct the miniaturized high‐throughput tissue array, which was completed by the Shanghai Outdo Biotech Company (Shanghai, China). The details of the patients are provided in Table S1. Ethical statement and ethics approval were obtained from Medical ethics committee of Changzhi People's Hospital, The Affiliated Hospital of Changzhi Medical College. The study was undertaken with the understanding and written consent of all subjects and in accordance with the Helsinki Declaration.

### 2.2 Tissue microarray

Tissue microarray (TMA) of CRC (94 patients) were purchased from the Shanghai Outdo Biotech Company. The specimens were diagnosed through immunohistochemical techniques, and all patients were categorized according to the seventh AJCC stage. The details of the patients are provided in Table S2. The Ethical statement was obtained from Ethics committee of Shanghai Outdo Biotech Company.

### 2.3 cBioPortal database analysis

The relationship between SMARCA4 and NOTCHs or SMARCA4, NOTCH3, MUC5AC, and MUC2 from 3806 patients/3953 samples of CRC in 10 studies was determined using the cBioPortal (https://www.cbioportal.org/) website of cancer genomics datasets [40]. The mutual exclusivity between SMARCA4 and NOTCH families in CRC was analyzed using the cBioPortal tool.

### 2.4 GeneMANIA and STRING databases

The biological network of NOTCH3 and SMARCA4 was constructed using GeneMANIA (http://genemania.org/) and STRING (https://string‐db.org/). GeneMANIA, an internet application, was used to build a biological network for NOTCH3 and SMARCA4 in the field of protein and phylogenetic relationship, pathways, co‐expression, co‐localization, prediction, and protein domain similarity, as well as to evaluate the functions of different components of the network [41]. Interactions were illustrated using a diagram wherein the nodes represent genes and the connections represent the networks. STRING is a database that provides information about the known and predicted PPIs. Direct (physical) and indirect (functional) correlations resulting from computer prediction, information transmission across species, and interactions collected from other (primary) databases were among the evaluated interactions [42].

### 2.5 Gene set enrichment analysis of NOTCH3 and SMARCA4

Gene set enrichment analysis (GSEA) was performed as reported earlier [43, 44]. The gsea software was downloaded from http://software.broadinstitute.org/gsea/index.jsp. The GSEA analysis was conducted on a large cohort TCGA dataset and by dividing the samples into two groups according to the median expression of NOTCH3. The C2 (c2.DELASERNA_TARGETS_OF_MYOD_AND_SMARCA4.gmt; c2.HENDRICKS_SMARCA4_TARGETS_UP.gmt; c2.LIU_SMARCA4_TARGETS.gmt; c2.MEDINA_SMARCA4_TARGETS.gmt) sub‐collection (http://www.gsea‐msigdb.org/gsea/msigdb/genesets.jsp.) obtained from the Molecular Signatures Database (http://software.broadinstitute.org/gsea/msigdb/index.jsp) were used as the reference gene sets. The default setting was performed, and the threshold significance was determined using permutation analysis (1000 permutations). The Enrichment Score (ES) and False Discovery Rate (FDR) were calculated. An FDR score < 0.25 and *P* < 0.05 were considered to indicate significant enrichment.

### 2.6 Immunohistochemical (IHC) analysis

The expression patterns of NOTCH3 and SMARCA4 in CRC and the paracancerous tissues were analyzed by IHC testing. IHC staining for NOTCH3 (ab23426; Abcam, Cambridge, UK) and SMARCA4 (21634‐1‐AP; Proteintech, Rosemont, IL, USA) was performed on the CRC consecutive sections of tissue arrays. The paraffin‐embedded tissue arrays were dewaxed in xylene for 15 min with the Leica ST5020 Automatic Staining machine (Leica Biosystems, Nussloch, Germany), hydrated in graded ethanol (100% ethanol for 7 min; 90% for 5 min; 80% for 5 min; 70% for 5 min), and sealed with 3% hydrogen peroxide (H_2_O_2_) for 10 min at room temperature to inactivate the endogenous peroxidase activity. The sample was washed thrice with 1 × PBS for 3 min each time, then sealed with 5% BSA at room temperature for 20 min. The tissue arrays were incubated with primary antibody at 4 °C overnight and then washed three times with 1 × PBS. The second antibody was incubated at 37 °C for 30 min, then washed thrice with 1 × PBS. The sections were incubated with horseradish peroxidase complex for 30 min at 37 °C and visualized with diaminobenzidine (DAB). All IHC images were collected by Olympus B × 51 microscopes (Olympus, Tokyo, Japan) and DP50 camera (Olympus).

In the tissue sections, the cells stained dark brown, brown‐yellow, and light yellow were strongly positive, moderately positive, and weakly positive, respectively, while the cells stained blue were negative. The IHC results were analyzed by the histological scoring method (*H*‐score). *H*‐score or the histochemistry score is obtained by conducting a histological scoring method for dealing with the results of immunohistochemistry, which converts the number of positive cells and their staining intensity into corresponding values in each section to achieve the purpose of semi‐quantitative tissue staining. *H*‐score = ∑(PI × *I*) = (percentage of cells of weak intensity × 1) + (percentage of cells of moderate intensity × 2) + (percentage of cells of strong intensity × 3), where PI represents the percentage of positive cells as a percentage of all cells in the section and *I* represent the staining intensity.

The staining of NOTCH3, SMARCA4, MUC5AC, and MUC2 in the TMAs of 94 clinical patient specimens was assessed by the Opal 7‐color Manual IHC Kit (PerkinElmer, Akron, OH, USA; NEL811001KT) and collected by the Vectra Polaris Automated Quantitative Pathology Imaging System (PerkinElmer). The specific antibody information is provided in Table S3.

### 2.7 Cell culture and transfection

Human colon cancer HT29, SW480, SW620, and HCT116 cell lines were derived from ATCC (Manassas, VA, USA). SW480 and SW620 were cultured in Leibovitz's Lmur15 medium (Gibco, Grand Island, NY, USA) and McCoy's 5a modified medium (Gibco) as the medium for HT29 and HCT116 cells. Penicillin (100 U·mL^−1^; Solarbio, Beijing, China), streptomycin (100 μg·mL^−1^; Solarbio), and heat‐inactivated 10% fetal bovine serum (Gibco) were added to all media before cell culture. The fusion rate of cells cultured at 37 °C under 5% CO_2_ atmosphere and 95% humidity was > 80%. These cells were harvested as described in the next section. The specific small‐interfering RNA (siRNA) of human NOTCH3 and SMARCA4 were constructed and synthesized by OBiO Technology (Shanghai, China) Co., Ltd. The NOTCH3 (NM_000435.3) intracellular domain (NICD3) over‐expression plasmid was constructed by OBiO Technology (Shanghai) Co., Ltd. The specific siRNA of NOTCH3 and SMARCA4 were transfected into the colon cancer cell line by Lipofectamine 3000 (Invitrogen Life Technologies, Chicago, IL, USA), according to the instructions. The siRNA‐specific sense strands for human NOTCH3 and SMACCA4 are shown in Table S4. The related verification reports of the knock‐down assays have been illustrated in Fig. S1.

### 2.8 Western blotting

The cells were lysed with RIPA buffer (C1053; Applygen Technologies, Beijing, China) containing protease inhibitor cocktail (Roche, Basel, Switzerland) and phenylmethane‐sulfonyl fluoride (PMSF) (Sigma, Milwaukee, WI, USA), and the total proteins were extracted. BCA protein assay kit (Thermo Scientific, Waltham, MA, USA) was used for the determination of protein concentration. According to the 1 : 4 ratio, 5 × loading buffer was added to boil denaturation. The protein samples were separated taking account of the molecular weight of the protein of interest by using 4–20% SDS/PAGE with the SDS/PAGE Running Buffer (B1005; Applygen Technologies) and then transferred onto polyvinylidene fluoride (PVDF) membranes. Thereafter, the membranes were sealed with 5% skimmed milk powder for 1 h and then incubated with the primary antibody overnight at 4 °C, followed by incubation with goat anti‐rabbit or goat anti‐mouse IgG antibody (Cell Signal Technology, Danvers, MA, USA) containing horseradish peroxidase at the room temperature for 1 h. Finally, the enhanced chemiluminescence solution was imaged by the AI600 image system, and β‐actin (Cell Signaling Technology) was used as the unified internal reference.

### 2.9 Coimmunoprecipitation assay

The colon cancer cells or HEK293T cells expressing SMARCA4‐GFP overexpression adenovirus (constructed from Novobio Scientific, Shanghai, China) and/or NICD3‐FLAG (OBiO Technology) were lysed with RIPA buffer (C1053; Applygen Technologies) containing protease inhibitor cocktail (Roche) and PMSF (Sigma) for the extraction of total proteins. The supernatant was collected after centrifugation at 12 000 ***g*** at 4 °C for 10 min, and the BCA Protein Assay Kit (Thermo Scientific) was used to determine the protein concentration. Following this step, 100 μL of the sample was used as the input and the other sample for the subsequent coimmunoprecipitation (co‐IP).

The supernatant of CRC cell lysate was incubated with 1.0 μg rabbit or mouse IgG (common IgG of the same origin as the antibody used in the IP experiment) and 20 μL of Protein G immunoprecipitation beads at 4 °C for 1 h, followed by centrifugation at 2000 ***g*** for 5 min at 4 °C. Then, the supernatant was taken and incubated with anti‐NOTCH3 (5276S; Cell Signaling Technology) or anti‐SMARCA4 (52251S; Cell Signaling Technology) antibody at the ratio of 1 : 50. The dilution ratios of anti‐Flag and anti‐GFP antibodies for the extraction of protein from transfected HEK293T cells are listed in Table S3. Then, 80 μL of protein A/G immunoprecipitation beads (mixed before use) were added to the mixture and incubated at 4 °C for 3–5 h on the rotary table. The immunoprecipitated complex was collected, followed by extended washing with 1 mL of pre‐cooled IP lysate (without adding various inhibitors) on the rotary table. Finally, 60 μL of 1 × loading buffer was added and boiled on a metal shaker at 99 °C for 10 min. Rabbit (B900610; Proteintech) or mouse (B900620; Proteintech) IgG (IgG‐IP) were used as negative controls. The specific antibody information is shown in Table S3.

### 2.10 Glutathione *S*‐transferase pull‐down assay

Two purified recombinant human glutathione *S*‐transferase (GST)‐tagged SMARCA4 segments [amino acids (aa) 612–656 and (aa) 1455–1566] (GeneCreate Biological Engineering Co., Ltd., Wuhan, China) and His‐tagged human NOTCH3 intracellular domain (aa 1665–2321) (GeneCreate Biological Engineering Co., Ltd.) were mixed and incubated on an ice bath for 3 h. Subsequently, the mixture was incubated with Streptavidin Magnetic Beads (New England Biolabs, Lpswich, MA, USA). After washing five times with the washing buffer, the proteins were eluted with the wash buffer supplemented with 15 mm of reduced glutathione. The elutes were separated using 12% SDS/PAGE and transferred onto the PVDF membranes (Millipore, Billerica, MA, USA), and then finally probed with anti‐GST (Proteintech) and anti‐His (Proteintech) antibodies. GST from Wuhan Genecreate (Wuhan, China) was used as the negative control.

### 2.11 CCK‐8 detection assay

The following experiments were carried out according to the instructions of the CCK‐8 cell proliferation and cytotoxicity detection kit (CA1210; Solarbio). The cells were inoculated into 96‐well plates. Different groups of cells were pretreated according to the experimental conditions. Cells were incubated in a 37 °C, 5% CO_2_ incubator for the indicated times (0, 24, 48, and 72 h). After adding 10 μL of CCK‐8 solution to each well, the cells were incubated in an incubator for 30 min. The medium without cells and the CCK‐8 solution group were used as blank controls. Finally, the absorbance at 450 nm was measured with a microplate reader (BioTek, Winusky, VT, USA).

### 2.12 Clone formation assay

The colon cancer cells were transfected in groups for 24 h, and the clone formation experiment was performed. For this purpose, 2000 cells were inoculated into each well of a six‐well plate, with three in each group, and the liquid was changed every 2–3 days. Two weeks later, clone formation was calculated. The supernatant was discarded, and the cells were washed twice with 1 × PBS for 10 min and then fixed with paraformaldehyde. Then, the fixed solution was discarded and dyed for 10 min with an appropriate amount of crystal violet, followed by 1 × PBS cleaning, counting, and taking pictures. Clone formation in this assay is calculated as follows:

$\text{Clone Formation Rate}\;\;\text{(}\%\text{)}=\left(\text{the number of clones}/\text{the number of inoculated cells)}\times 100\%\right.$

### 2.13 Transwell invasion and migration assay

After 36–48 h of transfection, Transwell analysis was performed in a modified Boyden chamber equipped with 8‐μm pore filters. The invasion assay was coated with Matrigel and then thawed overnight at 4 °C and diluted to obtain a final concentration of 1 mg·mL^−1^ with a serum‐free medium, followed by pre‐cooling at 4 °C on an ice bath. At the center of the bottom of the chamber, 100 μL of diluted Matrigel was added vertically and then incubated at 37 °C for 4–5 h to make it dry and gelatinous. The CRC cells (1 × 10^4^ cells/well) were treated under different conditions in the upper chamber, and the cell culture medium was added to the lower chamber, followed by incubation with 5% CO_2_ at 37 °C and under 95% humidity for 24 h, stained with crystal violet, and finally fixed. The cells from randomly selected fields were counted under the microscope. The procedure for the invasion and migration assay was repeated thrice.

### 2.14 Wound healing assay

The cells from different treatment groups were inoculated at an equal density into six‐well plates (5 × 10^5^ cells/well) in triplicate. A few cells were scratched from the center of each well with a small sterile pipette tip. The collected cells were gently washed with 1 × PBS thrice, the scribed cells were removed, and a fresh culture medium was added. The cells were incubated at 37 °C under a 5% CO_2_ humidified chamber for 48 h. The wound areas were visualized under a microscope (Nikon ECLIPSE 80i, Tokyo, Japan) at different time points (0, 24, and 48 h, respectively). Wound closure was then calculated using the following formula: Wound width (%) = (the scratch area at the ending point/the initial scratch area) × 100%. The procedure for the wound healing assay was repeated thrice.

### 2.15 Flow cytometry apoptosis detection assay

The following experiments were carried out according to the instructions of the Annexin V‐FITC/PI Apoptosis Detection Kit (CA1020; Solarbio). The cells were inoculated into six‐well plates (5 × 10^5^ cells/well). Different groups of cells were pretreated according to experimental conditions. After discarding the medium, trypsinize the cells with EDTA‐free trypsin, add cell culture medium to terminate the digestion, and collect the digested cells into a centrifuge tube. The cells were centrifuged at 106 ***g*** for 5 min, and the supernatant was discarded. Add 1 mL of 4 °C pre‐chilled 1 × PBS to resuspend the cells, and repeat the centrifugation step once. Dilute the Binding Buffer with deionized water at a 1 : 9 dilution ratio. Resuspend cells in 1 × binding buffer to maintain a cell concentration of 1–5 × 10^6^ cells·mL^−1^. Then take 100 μL of cell suspension and 5 μL Annexin V/FITC solution, mix well, and incubate at room temperature for 5 min in the dark. Finally, 5 μL of propidium iodide solution (PI, CA1020; Solarbio) and 400 μL of PBS were added and mixed, and flow cytometry was performed immediately.

### 2.16 Deep‐sequencing of RNA (RNA‐seq)

The total RNA of the sample was extracted and digested with deoxyribonuclease I (DNase I) (M0303L; New England Biolabs), then the mRNA was enriched with Oligo (dT) magnetic beads [Dynabeads™ Oligo (dT)_25_, 61005; Thermo Scientific] and broken into short fragments with the addition of the interruption reagent. The interrupted mRNA was used as a template to synthesize one‐strand cDNA, with six‐base random primers, and for the preparation of a two‐strand reaction system to synthesize two‐stranded cDNA, and the Agencourt AMPure XP kit (Beckman Coulter, Inc., Indianapolis, IN, USA) was used to purify double‐stranded cDNA. The purified double‐stranded cDNA was repaired, A‐tailed, and connected to the sequencing connector, and then the fragment size was selected, and finally, the PCR amplification was performed. After the constructed library passed the quality test of Agilent 2100 Bioanalyzer (Agilent Technologies Inc., Santa Clara, CA, USA), it was sequenced by Illumina HiSeqTM 2500 sequencer (Illumina, San Diego, CA, USA) to produce double‐terminal data of 125 or 150 bp. After passing the quality inspection, the Illumina sequencer was used for sequencing, and the sequencing data were obtained for follow‐up bioinformatics analysis.

### 2.17 Quantitative real‐time PCR

The total RNA was extracted from the CRC cells and transfected in different groups with the TRIzol reagent (Invitrogen Life Technologies). Quantitative real‐time PCR (qRT‐PCR) was performed with specific primers (Qiagen, Hilden, Germany) and the SYBR‐Green PCR Master Mix Kit (Takara, Tokyo, Japan) on the ABI 7500 System (Applied Biosystems, Foster City, CA, USA). GAPDH was used for normalizing the gene expression. All primers used in this study were synthesized by Sangong Biotech (Shanghai, China). The primers used in this study have been listed in Table S5.

### 2.18 Cellular immunofluorescence

The slides were soaked in 1 × PBS for 3 min, 4% paraformaldehyde was used to fix the slides for 30 min, and soak the slides for 3 min each time with 1 × PBS. PBS was sucked dry using an absorbent paper, and the slides were sealed with normal goat serum at room temperature for 30 min. After blocking, the cells were incubated with the corresponding primary antibodies mouse monoclonal anti‐MUC2 (ab11197; Abcam) and rabbit monoclonal anti‐MUC5AC (61193S; Cell signaling Technology) overnight at 4 °C, followed by treatment with Goat anti‐Mouse IgG (H+L) Cross‐Adsorbed‐Alexa Fluor 488 Secondary Antibody and Goat anti‐Rabbit IgG (H+L) Cross‐Adsorbed‐Alexa Fluor 594 Secondary antibody, respectively, for 1 h at room temperature, after which the sections were washed with 1 × PBS thrice for 3 min each time. The nucleus was stained with 4′, 6‐diamidinophenyl‐indole (DAPI) (D9542; Sigma). The sealing was performed using the sealing liquid containing an anti‐fluorescence quenching agent, and the slides were finally observed under a confocal microscope (TCS‐SP5; Leica, Mannheim, Germany).

### 2.19 Detection of MUC5AC and MUC2 in the HCT116 cell culture supernatant

The expression levels of MUC5AC and MUC2 in HCT116 cell culture supernatant were measured by ELISA for MUC5AC (Elabscience Biotechnology, Wuhan, China; E‐EL‐H2279c) and anti‐MUC2 (Novus Biologicals, Littleton, CO, USA; NBP2‐76700), respectively. The samples were prepared according to the manufacturers' instructions. Briefly, HCT116 cell supernatant was collected under each condition and centrifuged at 1000 ***g*** for 20 min at 4 °C. The samples or the standard working solution were added to the micro ELISA plate well and incubated at 37 °C for 90 min. After discarding the liquid from the plate, 100 μL of biotinylated antibody working solution was added immediately, mixed, and incubated at 37 °C for 60 min. After washing each well thrice with 350 μL of the washing solution, 100 μL of HRP conjugate working solution was added to each well, and the plate was incubated at 37 °C for 30 min. After discarding the liquid in the plate, each well was washed five times with the washing solution. Then, 90 μL of the substrate reagent was added to each well, and the samples were incubated at 37 °C for 15 min. After the stop solution was added, the optical density (OD value) of each well was measured at 450 nm using the SynergyH1 Microplate Reader (Biocompare, South San Francisco, CA, USA). The concentrations of MUC5AC and MUC2 were directly proportional to the OD value at 450 nm. Finally, the concentrations of MUC5AC and MUC2 in the sample were calculated by drawing the standard curve.

### 2.20 CHIP assay

The CHIP Assay Kit (56383; Cell Signaling Technology) was used to perform the CHIP assay as instructed by the manufacturer. Rabbit monoclonal anti‐NOTCH3 antibodies (5276S; Cell Signaling Technology) or rabbit anti‐IgG antibodies (B900610; Proteintech) were used for precipitation, and the IP was purified on protein A/G immunoprecipitation beads. The DNA purified from CHIP was ligated with the adapter and subjected to PCR amplification, as per the manufacturer's instructions (Illumina). We used the ConSite service based on the JASPAR datasets for this purpose. As is already known, the transcription factor CSL is the key effector of canonical NOTCH signaling. Accordingly, the transcription binding sites prediction of NOTCH3‐CSL to the *MUC5AC* and *MUC2* promoter were performed as described elsewhere [45]. The primer set used to amplify the promoter regions of *MUC5AC*: 5′‐GGAGGGAGAGTCTAGCCACA‐3′ (sense) and 5′‐GAAGCTGTTGACTGGTCCGA‐3′ (antisense); *MUC2* was: 5′‐CCTGTGTGCCTGATTCCGTA‐3′ (sense) and 5′‐GCTCATGGAGCTGTGTCAGA‐3′ (antisense). The specific antibody information is provided in Table S3.

### 2.21 Luciferase reporter assay

*MUC5AC* and *MUC2* promoter luciferase reporter plasmids containing the predictive binding sites of NOTCH3‐CSL were constructed with support from GeneCreate Biological Engineering Co., Ltd. The CSL/RBPJκ (CSL) overexpression plasmid was constructed from Hanheng (Shanghai, China). The two reporter plasmids were then co‐transfected with the CSL overexpression plasmid, NICD3 overexpression plasmid, and NC‐shRNA or SMARCA4‐shRNA into HCT116 cells using Lipofectamine™ 3000 (Thermo Scientific). At 48 h after transfection, firefly and renilla luciferase activities were measured using the Dual Luciferase@ Assay System (Promega, Madison, WI, USA). The luciferase activities were normalized by determining the renilla luciferase activity.

### 2.22 Statistical analysis

Statistical analyses were performed using the graphpad prism 6.01 Software (GraphPad Software, La Jolla, CA, USA). The data were expressed as mean ± standard errors of the mean (SEM). A two‐way analysis of variance (ANOVA) was used for multiple experimental group analyses. Differences between two groups were analyzed using Student's *t*‐test. The correlation analysis was evaluated by Pearson's correlation test. Survival analysis was estimated using the Kaplan–Meier method with the log‐rank test. *P* < 0.05 was considered to be statistically significant.

## 3 Results

### 3.1 Correlation between NOTCH3 and SMARCA4 based on database analysis in CRC

**Fig. 1:**
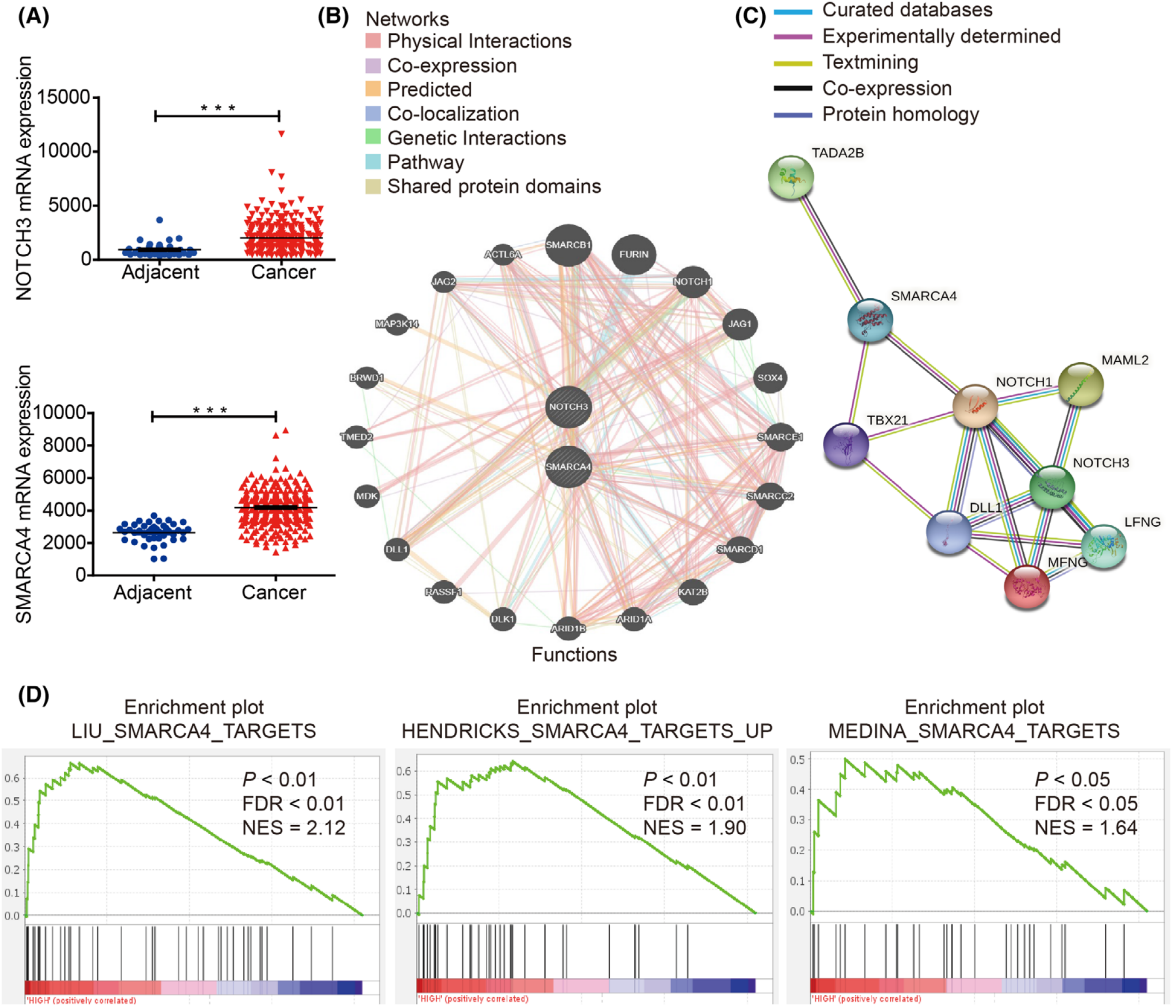
A close correlation was noted between NOTCH3 and SMARCA4 in multiple signaling pathways. (A) The expression of NOTCH3 and SMARCA4 in CRC tissues (*n* = 287) and the adjacent normal tissues (*n* = 41) based on the TCGA database. (B) The biological network between NOTCH3 and SMARCA4 through GeneMANIA (http://genemania.org/). (C) The biological network between NOTCH3 and SMARCA4 through STRING (https://string‐db.org/). (D) Enrichment plots of GSEA indicate that the gene signatures of SMARCA4 targets were significantly enriched in high NOTCH3‐expressing CRC specimens (*n* = 382). Data are presented as mean ± SEM; statistical analyses were performed using Student's *t* tests in A (***P < 0.001); FDR, false discovery rate; NES, normalized enrichment score. [Colour figure can be viewed at wileyonlinelibrary.com]

To explore whether SMARCA4 is involved in the regulation of the NOTCH signaling pathway in CRC. First, the correlation between SMARCA4 and NOTCH1, NOTCH2, NOTCH3, and NOTCH4 from 3806 patients/3953 samples from 10 studies based on the cBio Cancer Genomics Portal (http://cbioportal.org) were analyzed (Table 1). It was obvious that SMARCA4 and NOTCH families coexisted, with the closest coexistence between NOTCH3 and SMARCA4 (Log_2_ odds ratio > 3; *P* < 0.001; *q* < 0.001). TCGA database was used to analyze the expression of NOTCH3 and SMARCA4 in the CRC tissues and the adjacent normal tissues, and the results revealed that both of them showed higher expression in cancer tissues when compared to the adjacent tissues (Fig. 1A). The biological network between NOTCH3 and SMARCA4 was finally constructed using GeneMANIA (http://genemania.org/) and STRING (https://string‐db.org/) (Fig. 1B,C), which indicated a close relationship between NOTCH3 and SMARCA4 in multiple signaling pathways. In addition, GSEA analysis was also performed using the TCGA dataset, and, on calculating the pathway Enrichment Score (ES), it was found that the gene sets of LIU_SMARCA4_TARGETS, HENDRICKS_SMARCA4_TARGETS_UP, and MEDINA_SMARCA4_TARGETS were enriched by the higher expression of NOTCH3 (Fig. 1D) in CRC, which suggested that the high expression of NOTCH3 in CRC could activate the downstream signaling pathway of SMARCA4.

**Table 1:** The relationship between SMARCA4, NOTCH1, NOTCH2, NOTCH3, and NOTCH4 from 3806 patients/3953 samples in 10 studies (cBioPortal for Cancer Genomics).

A	B	Neither	A not B	B not A	Both	Log_2_ odds ratio	*P*‐Value	*q*‐Value	Tendency
SMARCA4	NOTCH3	3022	130	178	70	> 3	< 0.001	< 0.001	Co‐occurrence
SMARCA4	NOTCH1	3066	140	134	60	> 3	< 0.001	< 0.001	Co‐occurrence
NOTCH1	NOTCH3	3020	132	186	62	2.931	< 0.001	< 0.001	Co‐occurrence
NOTCH2	NOTCH3	3057	95	194	54	> 3	< 0.001	< 0.001	Co‐occurrence
SMARCA4	NOTCH2	3087	164	113	36	2.584	< 0.001	< 0.001	Co‐occurrence
NOTCH3	NOTCH4	3036	209	116	39	2.288	< 0.001	< 0.001	Co‐occurrence
NOTCH1	NOTCH4	3083	162	123	32	2.308	< 0.001	< 0.001	Co‐occurrence
NOTCH2	NOTCH4	3121	124	130	25	2.275	< 0.001	< 0.001	Co‐occurrence
SMARCA4	NOTCH4	3073	172	127	28	1.978	< 0.001	< 0.001	Co‐occurrence
NOTCH1	NOTCH2	3081	170	125	24	1.799	< 0.001	< 0.001	Co‐occurrence

### 3.2 NOTCH3 and SMARCA4 are closely related to the protein level in CRC

**Fig. 2:**
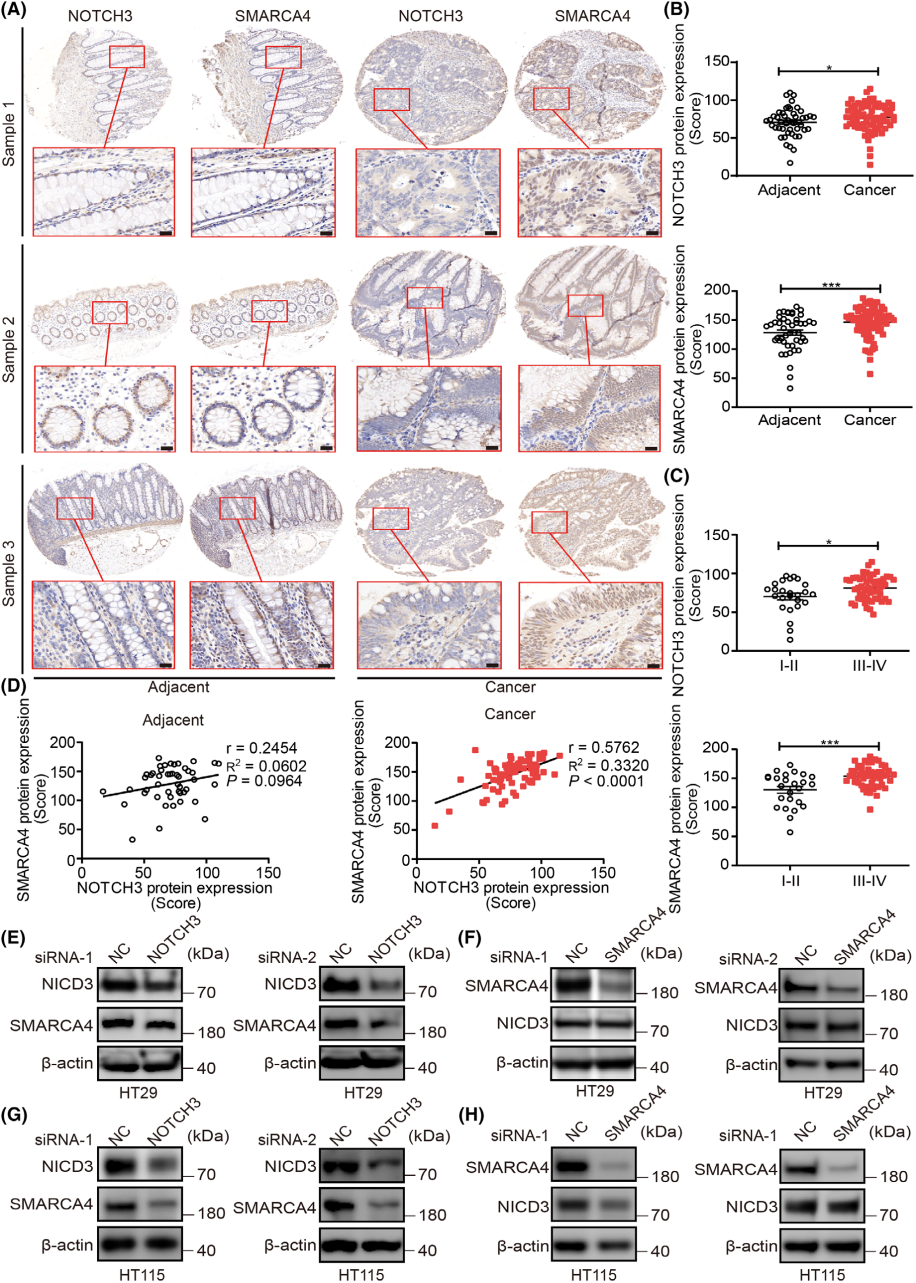
NOTCH3 and SMARCA4 are closely related at the protein level in CRC. (A) Representative images of IHC staining for NOTCH3 and SMARCA4 in human CRC (*n* = 79 chip points) and adjacent tissues (*n* = 47 chip points), (magnification, 80×, 200×). High‐magnification images of the boxed areas are shown in the inserts (ROI). Scale bar: 20 μm. (B) Semi‐quantitative results of NOTCH3 and SMARCA4 expression levels in adjacent (*n* = 47 chip points) and cancer tissues (*n* = 79 chip points). (C) Semi‐quantitative results of the NOTCH3 and SMARCA4 expression levels in stage I–II (*n* = 25 chip points) and III–IV (*n* = 54 chip points) cancer tissues. (D) The correlation between NOTCH3 and SMARCA4 in adjacent (*n* = 47 chip points) and cancer tissues (*n* = 79 chip points). (E) Both the targets of NOTCH3 siRNA can effectively inhibit the expression of NICD3 and reduce the expression of SMARCA4 in HT29 cells (*n* = 3). (F) Both the targets of SMARCA4 siRNA could effectively inhibit the expression of SMARCA4, but did not affect the expression of NICD3 in HT29 cells (*n* = 3). (G) Both the targets of NOTCH3 siRNA could effectively inhibit the expression of NICD3 and reduce the expression of SMARCA4 in HT115 cells (*n* = 3). (H) Both the targets of SMARCA4 siRNA could effectively inhibit the expression of SMARCA4, but did not affect the expression of NICD3 in HT115 cells (*n* = 3). Data are presented as mean ± SEM. Statistical analyses were performed using unpaired Student's *t* tests for B and C. Correlations were analyzed using Pearson's correlation test for D. *P < 0.05, ***P < 0.001; ns, no significant; NC, negative control; si, short interfering. [Colour figure can be viewed at wileyonlinelibrary.com]

To further clarify the correlation between NOTCH3 and SMARCA4, IHC was performed using the CRC and paracancerous tissue array to explore the correlation between NOTCH3 and SMARCA4. The results suggested that the expression of NOTCH3 and SMARCA4 in CRC tissues was significantly higher than that in the paracancerous tissues at the protein level, this difference was statistically significant (Fig. 2A,B). Moreover, the expression of NOTCH3 and SMARCA4 were increased at tumor progression stages III and IV relative to that in the tumor progression stages I and II (Fig. 2C). Further analysis of the correlation between NOTCH3 and SMARCA4 proteins in the CRC tissues and adjacent tissues revealed a significant positive correlation between them in the cancer tissues, but not in the adjacent tissues (Fig. 2D). In addition, prognostic model analysis was performed using the study cohort of patients with CRC and the TCGA database. The data revealed that the expression patterns of NOTCH3 and SMARCA4 proteins were linked to the prognosis of patients with CRC (Fig. S2).

To investigate the common features of the signaling pathway regulation between NOTCH3 and SMARCA4 in colon cancer cells, the CRC HT29 and HT115 cells (according to the relative mRNA expression of NOTCH3 in different CRC cells, cell lines with higher NOTCH3 transcriptional expression were selected; Fig. S1A) were used to knockdown NOTCH3 or SMARCA4 by transfecting the specific NOTCH3‐siRNAs or SMARCA4‐siRNAs, respectively. WB analysis revealed that both NOTCH3‐siRNAs and SMARCA4‐siRNAs could effectively knock down the expressions of NOTCH3 and SMARCA4 in HT29 cells (Fig. 2E,F) and HT115 cells (Fig. 2G,H), respectively. Subsequently, NOTCH3 knockdown was observed to significantly reduce the expression of SMARCA4 in HT29 cells (Fig. 2E). However, the knockdown of SMARCA4 did not affect the expression of NOTCH3 in HT29 cells (Fig. 2F). When NOTCH3 or SMARCA4 were knocked down, the results in HT115 cells were consistent with those in HT29 cells (Fig. 2G,H). Collectively, these results indicated that NOTCH3 may be involved in the progression of CRC by regulating SMARCA4, and further suggested a possible correlation between them in signal transduction. However, as the key transcriptional regulatory factor, whether SMARCA4 participates in the transcriptional regulation of NOTCH3 or whether they interact with each other during CRC remains unestablished.

### 3.3 NOTCH3 interacts with SMARCA4 directly in CRC cells

**Fig. 3:**
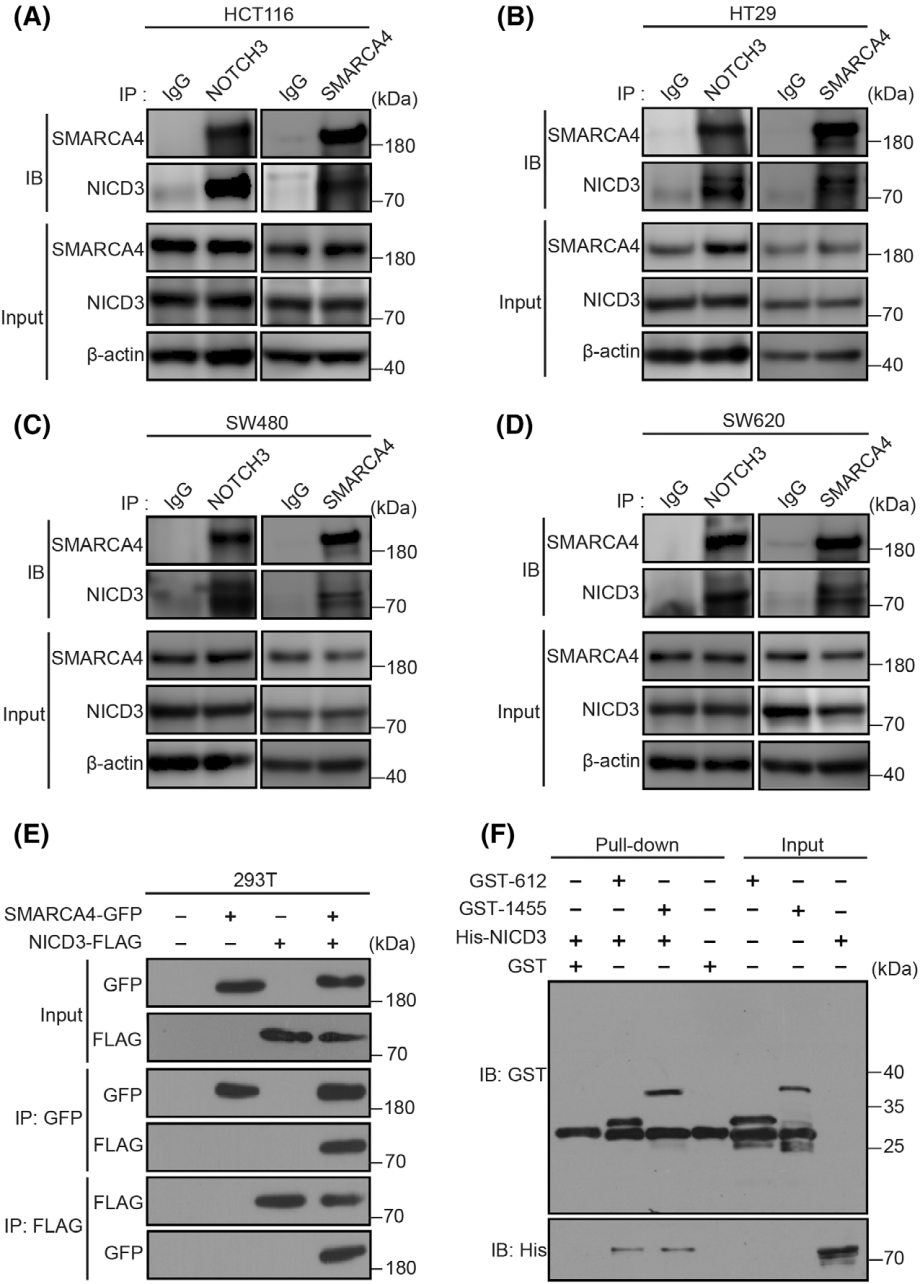
NICD3 interacts with SMARCA4 directly in CRC cells. (A) Endogenous co‐immunoprecipitation (IP) experiments performed on HCT116 cell lysates (*n* = 3). The immunoprecipitation assay of HCT116 cell lysates with NICD3, SMARCA4, or control (IgG) antibody. NICD3 or SMARCA4 was detected by WB using the indicated antibody. (B) Endogenous co‐IP experiments performed on HT29 cell lysates (*n* = 3). (C) Endogenous co‐IP experiments performed on SW480 cell lysates (*n* = 3). (D) Endogenous co‐IP experiments performed on SW620 cell lysates (*n* = 3). (E) Exogenous co‐IP experiments performed on lysates of 293T cells expressing NICD3‐FLAG and/or SMARCA4‐GFP (*n* = 2). The transfected constructs are indicated above. The antibodies used for IP and Western blotting (WB) analysis are shown on the left side. (F) Direct interaction between NICD3 and the two domains of SMARCA4 [SMART BRK domain (aa 612–656) and SMART BROMO domain (aa 1455–1566)] are depicted by the GST pull‐down assay (*n* = 2).

To determine whether NOTCH3 interacted with SMARCA4, endogenous co‐IP experiments were performed to explore the interactions between them in colon cancer cells. Different CRC cell lines were observed to illustrate the endogenous interaction between NOTCH3 and SMARCA4, and the results are depicted in Fig. 3A–D. In general, NOTCH3 was found to be coimmunoprecipitated with SMARCA4 in HCT116, HT29, SW480, and SW620 cells. Meanwhile, FLAG‐tagged NICD3 and/or GFP‐tagged SMARCA4 were/was overexpressed in the HEK293T cells for exogenous co‐IP experiments, which confirmed the interaction between recombinant NICD3 and SMARCA4 (Fig. 3E). Thereafter, a GST‐pulldown assay was performed to further determine whether there was a direct interaction between them. Because of the high molecular weight (185 kDa) of SMARCA4, its expression and purification *in vitro* are difficult. Therefore, SMART (http://smart.embl.de/) was used to predict the domains in which SMARCA4 may interact with other proteins. The results showed that SMARCA4 contained two domains (http://smart.embl.de/smart/show_motifs.pl?ID=SMCA4_HUMAN) that may interact with other proteins, namely the SMART BRK domain (aa 612–656) and SMART BROMO domain (aa 1455–1566). Furthermore, His‐NICD3, GST‐612 (BRK domain), and GST‐1455 (BROMO domain) were expressed and purified using *Escherichia coli* and subjected to GST pulldown assays. The results showed that His‐NICD3 was pulled down by GST‐612 and GST‐1455 (Fig. 3F), which indicated that SMARCA4 could interact directly with the intracellular domain of NOTCH3 (aa 1665–2321) via the BRK and BROMO domains, respectively.

### 3.4 NOTCH3 regulates the proliferation, invasion, and migration of colon cancer cells via the recruitment of SMARCA4

**Fig. 4:**
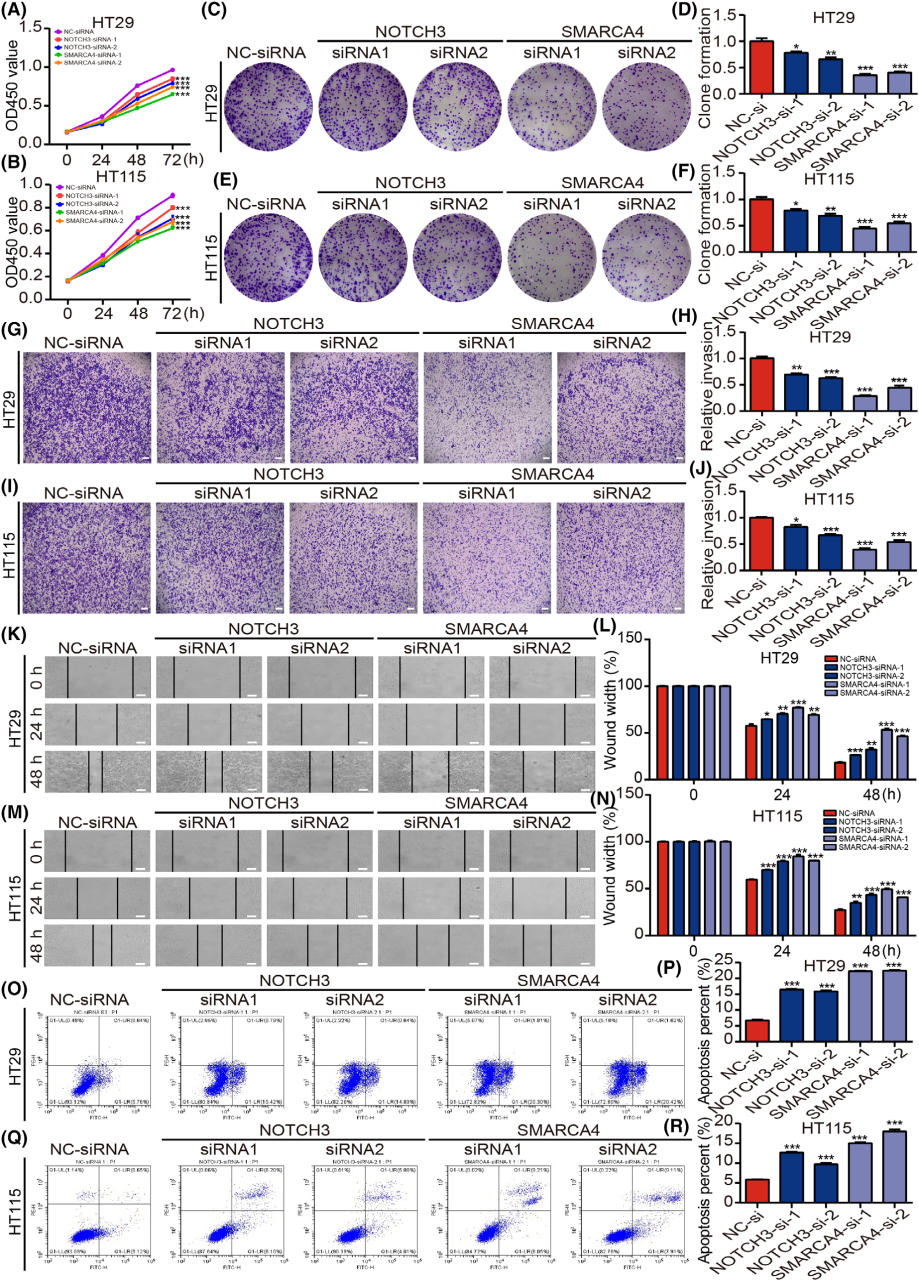
Both NOTCH3 and SMARCA4 can regulate the proliferation, invasion, migration, and apoptosis in colon cancer cells. (A, B) The CCK‐8 assay was performed to detect the proliferative activity of HT29 cells and HT115 cells in different groups (*n* = 3). (C–F) The effect of knocking down NOTCH3 or SMARCA4 on the ability of HT29 cells and HT115 cells' clone formation ability was detected by the clone formation assay and statistical analysis was performed (*n* = 3). (G–J) Transwell invasion assays were performed to detect the invasion abilities of HT29 cells and HT115 cells in different groups and the related statistical analysis was performed (scale bar: 100 μm; *n* = 3). (K–N) The scratch assays were performed to detect the migration abilities of HT29 cells and HT115 cells in different groups, and the related statistical analysis was performed (scale bar: 100 μm; *n* = 3). (O–R) Flow cytometry was performed to detect the apoptosis rate of HT29 cells and HT115 cells in different groups and its statistical analysis was performed (*n* = 3). Data are shown as presented as mean ± SEM. Statistical analyses were performed using two‐way ANOVA for CCK‐8 comparison in A and B. Multiple unpaired Student's *t*‐tests were using for the remaining data. *P < 0.05, **P < 0.01, ***P < 0.001; ns, no significant; NC, negative control; si, short interfering. [Colour figure can be viewed at wileyonlinelibrary.com]

NICD3 was observed to regulate and directly interact with SMARCA4 in CRC cells. To investigate the common features in the signaling pathway regulation of both NOTCH3 and SMARCA4 in CRC cells, the CRC HT29 and HT115 cells were used to knock down NOTCH3 and SMARCA4 by transfecting specific siRNAs, respectively. First, the proliferative activity of HT29 and HT115 cells was detected using the CCK‐8 assay (Fig. 4A,B), which showed that the activity decreased significantly after knockdown of NOTCH3 or SMARCA4. Furthermore, the alterations in the proliferation, invasion, and migration of HT29 and HT115 cells after knocking down NOTCH3 or SMARCA4 were detected using plate clone formation, transwell invasion, and wound healing assays, followed by the detection of apoptosis of HT29 and HT115 cells using flow cytometry. The above results confirmed that the clone formation, invasion, and migration abilities of both CRC cells decreased significantly after the knockdown of NOTCH3 or SMARCA4, and the difference was statistically significant (Fig. 4C–N), which revealed that both NOTCH3 and SMARCA4 are involved in the regulation of growth, invasion, and migration of CRC cells. For cell apoptosis, both the knockdown of NOTCH3 and SMARCA4 promoted the apoptosis of CRC cells to a certain extent (Fig. 4O–R).

**Fig. 5:**
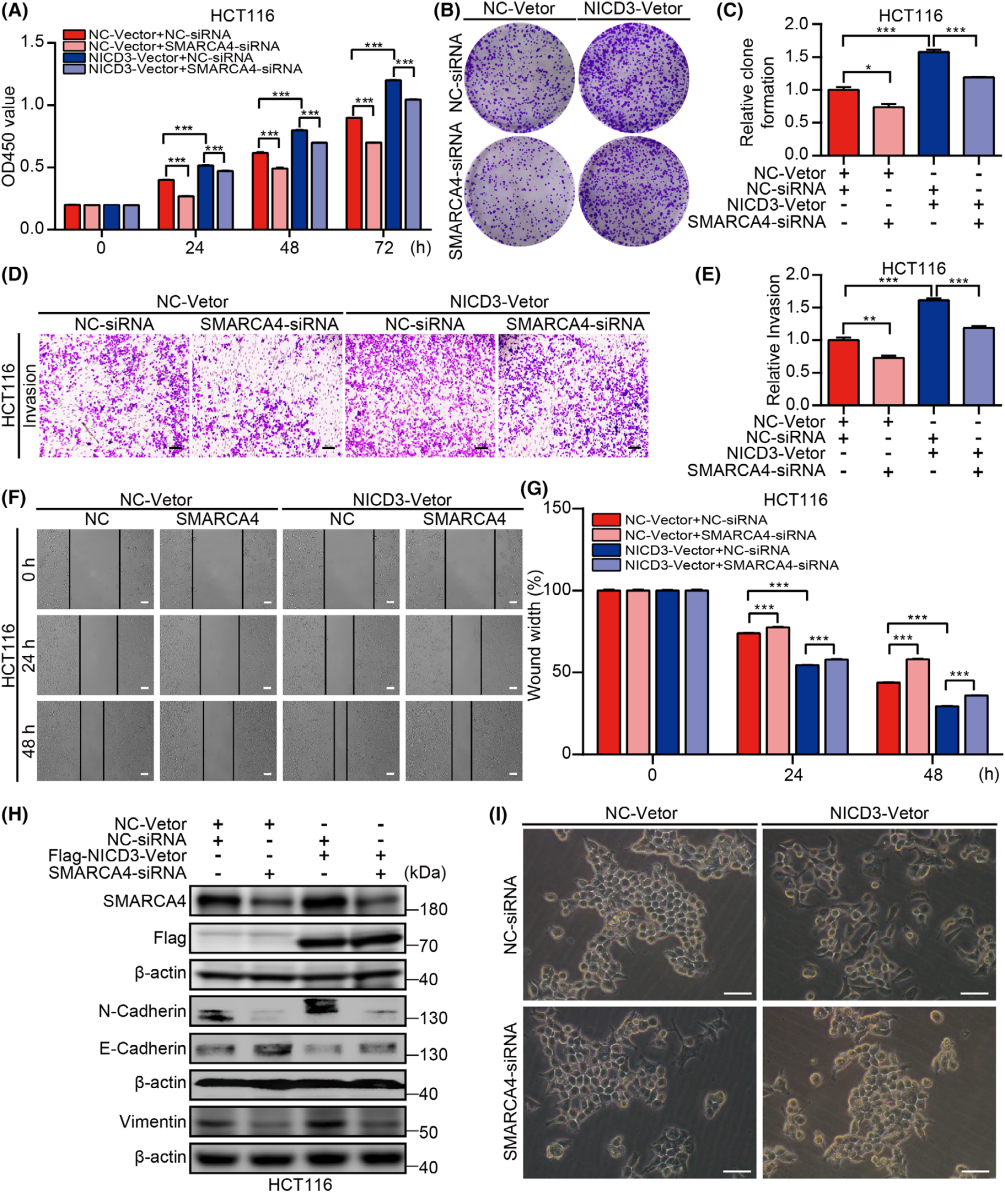
NOTCH3 regulates the progression of HCT116 cells in a SMARCA4‐dependent manner. (A) CCK‐8 assay was performed to detect the proliferative activity of HCT116 cells in different groups (*n* = 3). (B, C) The clone formation assay was used to detect the proliferative activity of HCT116 cells in different groups and related statistical analysis was performed (*n* = 3). (D, E) Transwell invasion assay was performed to detect the invasion abilities of HCT116 cells in different groups, and the related statistical analysis was performed (scale bar: 400 μm; *n* = 3). (F, G) The scratch assays were performed to detect the migration abilities of HCT116 cells in different groups, and the related statistical analysis was performed (scale bar: 100 μm; *n* = 3). (H) Knockdown of SMARCA4 increased the E‐cadherin expression and decreased the N‐cadherin and vimentin expression in NICD3‐overexpressed HCT116 cells (*n* = 3). (I) Morphological assays were performed to detect the morphological changes of HCT116 cells in different groups (scale bar: 25 μm; *n* = 5). Data are presented as mean ± SEM. Statistical analyses were performed using unpaired Student's *t* tests. *P < 0.05, **P < 0.01, ***P < 0.001; NC, negative control; si, short interfering. [Colour figure can be viewed at wileyonlinelibrary.com]

The above results suggest that NOTCH3 interacts with SMARCA4 to regulate the occurrence and development of CRC cells. To ascertain whether NOTCH3 was involved in the regulation of progression of CRC cells in a SMARCA4‐dependent manner, NICD3 was overexpressed and SMARCA4 was knocked down in the colon cancer cells. The proliferation, invasion, and migration capacities of CRC cells were then detected using CCK‐8, plate clone formation, transwell, and wound healing assays. The results signified that the overexpression of NICD3 significantly increased the proliferation, invasion, and migration abilities of CRC cells. On the contrary, the knockdown of SMARCA4 restored the increased CRC cells proliferation, invasion, and migration abilities induced by the overexpression of NICD3 (Fig. 5A–G, Fig. S3A,B). Epithelial–mesenchymal transformation (EMT) is an important biological mechanism by which the epithelial cancer cells gain migration and invasion abilities [46]. Reduced expression of epithelial markers, such as E‐cadherin, and increased expression of mesenchymal markers, such as N‐cadherin and vimentin, are three of the most noticeable alterations [47]. Therefore, the alterations in the expressions of EMT markers E‐cadherin, N‐cadherin, and vimentin were further explored. The overexpression of NICD3 via the simultaneous knockdown of SMARCA4 significantly decreased the expression of N‐cadherin and vimentin and increased the expression of E‐cadherin. The difference was statistically significant when compared with the single overexpression of NICD3 (Fig. 5H, Fig. S3C). Furthermore, how different treatments affected the morphology of HCT116 cells was examined. It is common knowledge that EMT refers to the transformation of epithelial cells from regular epithelial cells, such as rectangular, oblate, or cuboidal cells, which were closely arranged and regular, to irregular mesenchymal cells, such as spindle, shuttle, or star‐shaped cells. Accordingly, the results of the morphological assay confirmed that the mesenchymal characteristics of HCT116 cells were significantly increased after NICD3 overexpression. Moreover, this phenomenon was markedly reversed after knocking down SMARCA4 (Fig. 5I).

### 3.5 NOTCH3 interacts with SMARCA4 target to the expression of mucin MUC5AC and MUC2 by affecting their transcriptional activity

To elucidate the downstream targeting pathways or factors regulated by both NOTCH3 and SMARCA4 in colon cancer cells, bulk RNA‐seq was applied using HT29 cells transfected with NC‐siRNA, NOTCH3‐siRNA, and SMARCA4‐siRNA, respectively. Differentially expressed genes (DEGs) among the NC‐siRNA and NOTCH3‐siRNA or SMARCA4‐siRNA group were identified by DESeq2 [48]. *P* < 0.01 and fold change > 2 or fold change < 0.5 was set as the threshold for significant differential expression.

**Fig. 6:**
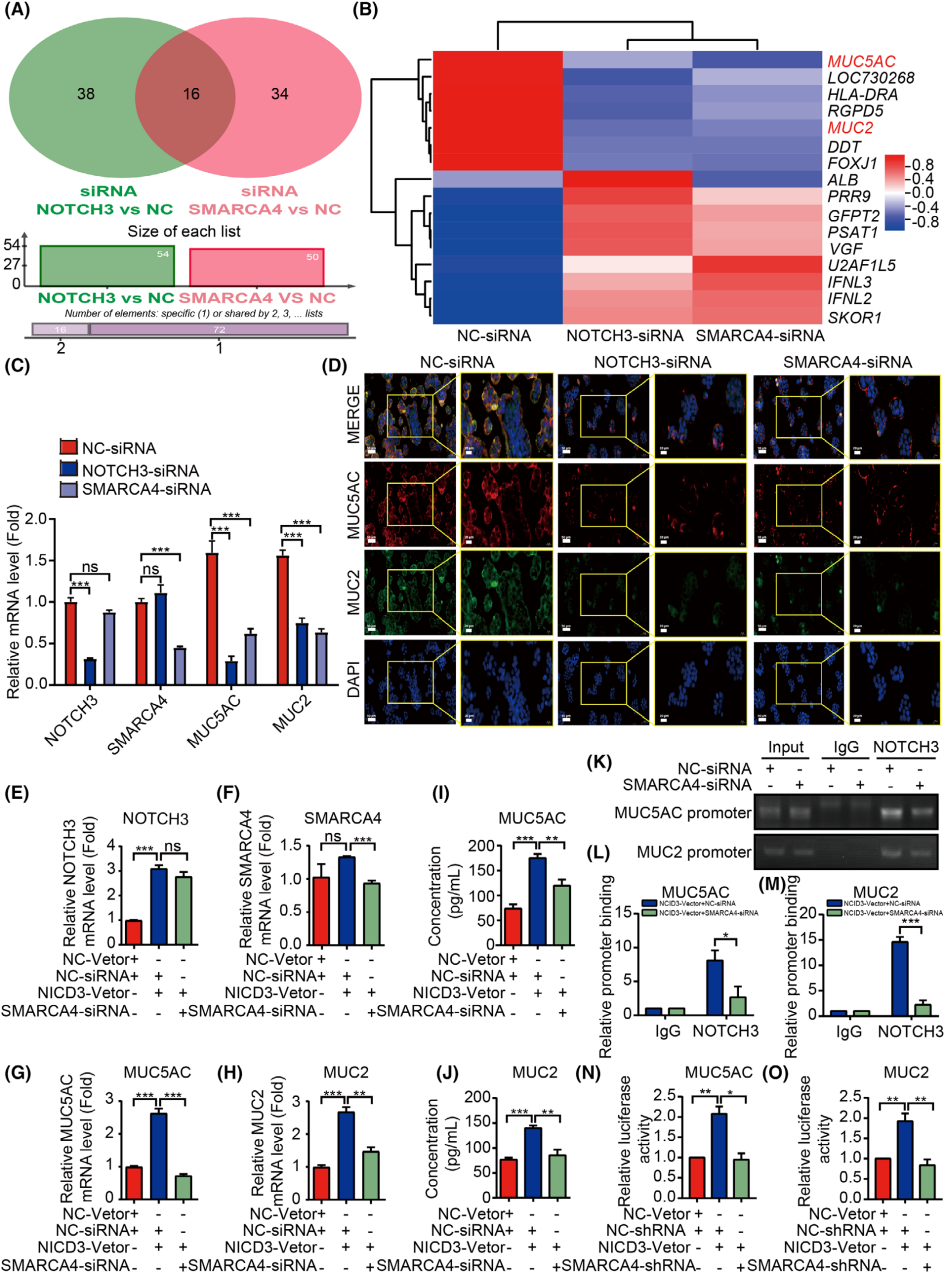
NOTCH3 interacts with the SMARCA4 target to the expression of MUC5AC and MUC2. (A) The Venn diagram depicts the DEGs and their intersection determined by RNA‐seq analysis after the knockdown of NOTCH3 or SMARCA4 in HT29 cells. (B) The 16 common genes were analyzed by heatmap cluster analyses. (C) The mRNA expression of NOTCH3, SMARCA4, MUC5AC, and MUC2 in different tissues by qRT‐PCR (*n* = 3). (D) The expression of MUC5AC and MUC2 proteins in different cell groups was detected by cellular immunofluorescence staining. Scale bar: 50 μm. High‐magnification images of the boxed areas are shown in the inserts (ROI, scale bar: 20 μm. *n* = 3). (E–H) Knockdown of SMARCA4 could partially restore the increased mRNA expression of MUC5AC and MUC2 caused by the NICD3 overexpression in HCT116 cells (mRNA by qRT‐PCR, *n* = 3). (I, J) The knockdown of SMARCA4 could partially restore the increased protein expression of MUC5AC and MUC2 caused by the NICD3 overexpression in HCT116 cells (protein by ELISA, *n* = 4). (K–M) The analysis of NOTCH3 binding to the MUC5AC and MUC2 promoter by ChIP assays in HCT116 cells transfected with either NICD3‐Vector + NC‐siRNA or NICD3‐Vector + SMARCA4‐siRNA (*n* = 3). Genomic DNA was immunoprecipitated with an anti‐NOTCH3 antibody, where IgG served as a negative control. The primer set specific to the NOTCH3‐binding site of MUC5AC and MUC2 promoter was applied in real‐time PCR amplification. (N, O) The luciferase activity of the MUC5AC and MUC2 promoter‐driven luciferase reporter was significantly increased by the transfected NICD3 plasmid (*n* = 3). The knockdown of SMARCA4 could partially restore the luciferase activity of MUC5AC and MUC2 promoter‐driven luciferase reporter caused by NICD3. Data are presented as mean ± SEM. Statistical analyses were performed using unpaired Student's *t* tests. *P < 0.05, **P < 0.01, ***P < 0.001; ns, no significant; NC, negative control; si, short interfering; sh, short hairpin. [Colour figure can be viewed at wileyonlinelibrary.com]

Compared with the NC‐siRNA group, the NOTCH3‐siRNA group had 54 differential genes meeting the above conditions, and the SMARCA4‐siRNA group had 50 differential genes meeting the above conditions. The intersection of these differential genes was considered, and 16 differential genes were reported from both groups (Fig. 6A). The 16 common genes were analyzed by heatmap cluster analysis (Fig. 6B). Among them, the decreased genes were *MUC5AC*, *LOC730268*, *HLA‐DRA*, *RGPD5*, *MUC2*, *DDT*, and *FOXJ1*, and the increased genes were *PRR9*, *GFPT2*, *PSAT1*, *VGF*, *U2AF1L5*, *IFNL3*, *IFNL2*, and *SKOR1*, while the expression of *ALB* increased after the NOTCH3 knockdown and decreased after the SMARCA4 knockdown. In recent years, several studies have shown that mucins play an important regulatory role in the progression of CRC, especially MUC2 and MUC5AC [3, 49, 50, 51, 52]. Moreover, the molecular evaluation revealed that the overexpression of MUC5AC and MUC2 proteins is one of the most obvious molecular features that distinguish MCA from non‐MCA [3, 36, 37, 38, 39]. This finding may suggest that the interaction between NOTCH3 and SMARCA4 plays a key regulatory role in the occurrence and development of MCA. For further verification, the changes in the MUC5AC and MUC2 expression at the mRNA and protein levels in HT29 cells by knocking down NOTCH3 or SMARCA4, respectively, were detected. The results revealed that, after knocking down NOTCH3 or SMARCA4, the mRNA and protein expression levels of MUC5AC and MUC2 significantly decreased, and the difference was statistically significant (Fig. 6C,D).

To demonstrate that NOTCH3 regulates the expression of MUC5AC and MUC2 by recruiting SMARCA4, the expression of SMARCA4 was inhibited when overexpressing NICD3 in HCT116 cells. After the overexpression of NICD3 in HCT116 cells, the mRNA and protein expressions of MUC5AC and MUC2 revealed a significant increase, as detected by qRT‐PCR and ELISA assays, while the overexpression of NICD3 and the simultaneous inhibition of SMARCA4 could significantly reduce the mRNA and protein expressions of MUC5AC and MUC2 (Fig. 6E–J). The difference was statistically significant as compared to the simple overexpression of NICD3. To further examine whether NOTCH3 regulated the expressions of MUC5AC and MUC2 through transcriptional activity by recruiting SMARCA4, we performed CHIP‐qPCR assays. The classical NOTCH signaling pathway is also known as the CSL‐dependent pathway. Accordingly, we first predicted that the transcription binding sites of NOTCH3‐CSL to the *MUC5AC* and *MUC2* were promoted through the JASPAR datasets and then verified the same by CHIP‐qPCR experiments. We discovered that NOTCH3 could bind to the *MUC5AC* and *MUC2* promoter fragments that included the CSL binding sites using CHIP assays (Fig. 6K–M). Hence, SMARCA4 depletion may prevent NOTCH3 from binding to the *MUC5AC* and *MUC2* promoters (Fig. 6K–M).

In addition, we designed the *MUC5AC* and *MUC2* promoter luciferase reporter plasmids, and the CSL overexpression plasmid for use in a dual‐luciferase reporter assay, which revealed that the luciferase activity of the *MUC5AC* and *MUC2* promoter‐driven luciferase reporter was significantly increased by the transfected NICD3 plasmid, while the knockdown of SMARCA4 could partially restore the luciferase activity of *MUC5AC* and *MUC2* promoter‐driven luciferase reporter caused by NICD3 (Fig. 6N,O). In summary, accumulated evidence indicated that SMARCA4 contributes to the recruitment of NOTCH3 to the *MUC5AC* and *MUC2* promoters. Moreover, a reversal experiment confirmed that the knockdown of MUC5AC or MUC2 could inhibit the ability of cell migration and invasion in CRC cells with the overexpression of NICD3 or SMARCA4 (Fig. S4). Therefore, NOTCH3 may participate in the differentiation and development of MCA by regulating the expression of *MUC5AC* and *MUC2*, which is dependent or at least partially dependent on SMARCA4.

### 3.6 Expression of NOTCH3 and SMARCA4 in CRC differentiated by MUC5AC/2

**Fig. 7:**
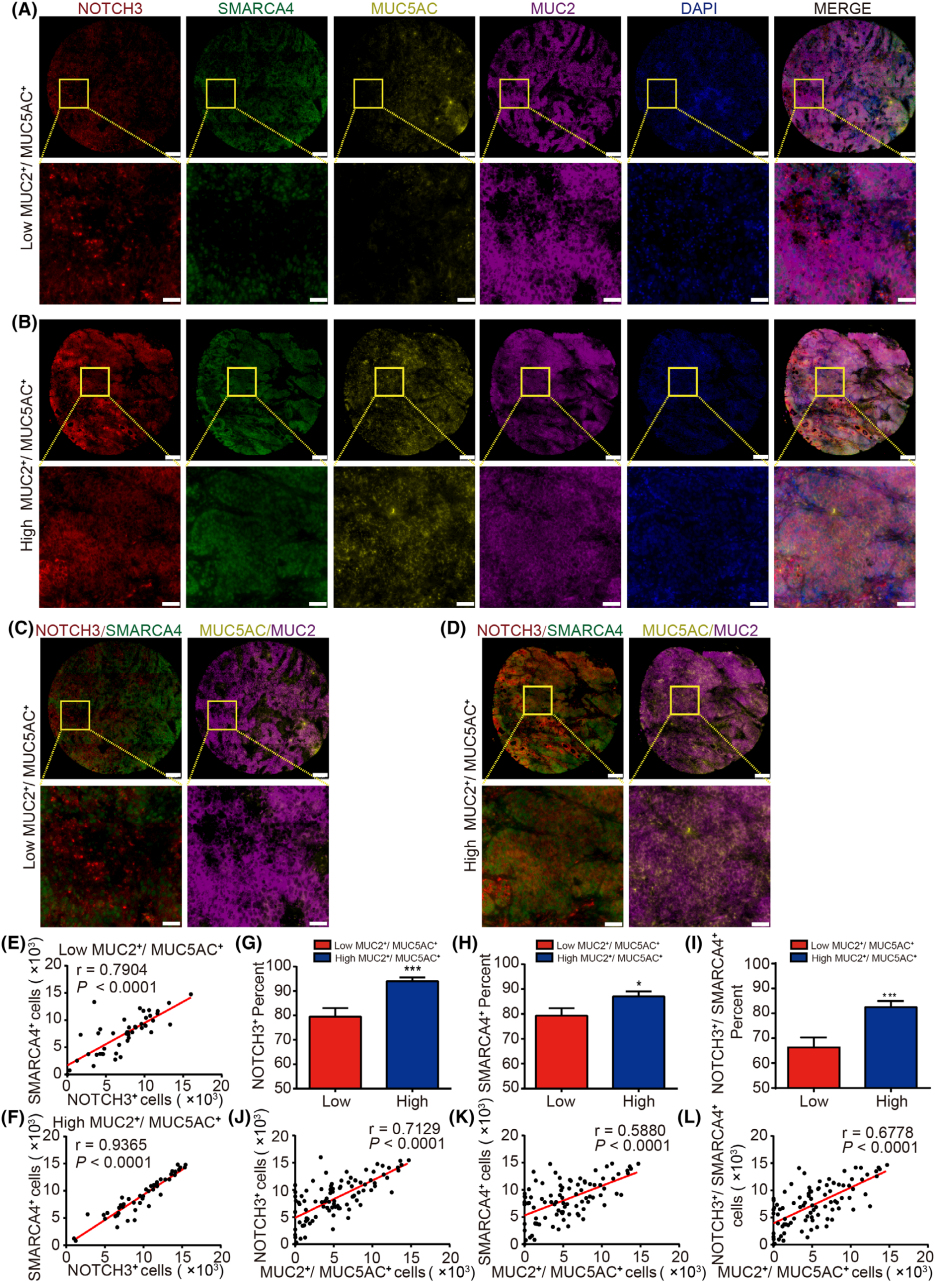
Expression of NOTCH3 and SMARCA4 in CRC differentiated by MUC5AC/2. (A) Representative images of IHC staining for NOTCH3, SMARCA4, MUC5AC, and MUC2 in the poor expression of the MUC5AC and MUC2 group (*n* = 47). (B) Representative images of IHC staining for NOTCH3, SMARCA4, MUC5AC, and MUC2 in the high co‐expression of the MUC5AC and MUC2 group (*n* = 47). (C) Representative images of IHC staining for NOTCH3 and SMARCA4 in the poor expression of the MUC5AC and MUC2 group (*n* = 47). (D) Representative images of IHC staining for NOTCH3 and SMARCA4 in the high co‐expression of the MUC5AC and MUC2 group (*n* = 47). (E) The correlation between NOTCH3 and SMARCA4 in CRC patients with the poor co‐expression of MUC5AC and MUC2 (*n* = 47). (F) The correlation between NOTCH3 and SMARCA4 in CRC patients with the high co‐expression of MUC5AC and MUC2 (*n* = 47). (G) The positive rate of cells expressing NOTCH3 was markedly higher in the MUC5AC and MUC2 co‐overexpression positive group (positive group: *n* = 47; negative group: *n* = 47). (H) The positive rate of cells expressing SMARCA4 was markedly higher in the MUC5AC and MUC2 co‐overexpression positive group (positive group: *n* = 47; negative group: *n* = 47). (I) The positive rate of cells co‐expressed with NOTCH3 and SMARCA4 was markedly higher in the MUC5AC and MUC2 co‐overexpression positive group (positive group: *n* = 47; negative group: *n* = 47). (J) The positive correlation between the number of MUC5AC and MUC2 co‐expressed positive cells and the number of NOTCH3‐positive cells (*n* = 94). (K) The positive correlation between the number of MUC5AC and MUC2 co‐expressed positive cells and the number of SMARCA4 positive cells (*n* = 94). (L) The positive correlation between the number of MUC5AC and MUC2 co‐expressed positive cells and the number of NOTCH3 and SMARCA4 co‐expressed positive cells (*n* = 94). Scale bar = 200 μm. High‐magnification images of the boxed areas are shown in the inserts (ROI). Scale bar: 50 μm. Data are presented as mean ± SEM. Statistical analyses were performed using unpaired Student's *t*‐tests. Correlations were analyzed using Pearson's correlation test. *P < 0.05, ***P < 0.001. [Colour figure can be viewed at wileyonlinelibrary.com]

The correlation among NOTCH3, SMARCA4, MUC5AC, and MUC2 from 3806 patients/3953 samples from 10 studies based on the cBio Cancer Genomics Portal (http://cbioportal.org) was further analyzed (Table 2). It was found that there was an obvious correlation among NOTCH3, MUC5AC, and MUC2, as well as among SMARCA4, MUC5AC, and MUC2. To further clarify the correlation between them at the tissue level, we performed multicolor fluorescence immunohistochemistry to detect the human CRC tissue samples for the expression of multiple markers (Fig. 7A–D). The increased expression of MUC5AC and MUC2 is one of the most obvious molecular features of MCA [3, 36, 37, 38, 39]. Therefore, the positive rate of cells co‐expressed with MUC5AC and MUC2 was used to classify the tumors, and the correlational analysis revealed a higher positive correlation between NOTCH3 and SMARCA4 in CRC patients with the high co‐expression of MUC5AC and MUC2 when compared with the low co‐expression of MUC5AC and MUC2 (Fig. 7E,F). In addition, the positive rate of cells expressing NOTCH3, SMARCA4, and co‐expressed with NOTCH3 and SMARCA4 was markedly higher in the high co‐expression positive group of the MUC5AC and MUC2 (Fig. 7G–I). Further analysis revealed a positive correlation between the positive rate of cells co‐expressed with MUC5AC and MUC2 and that the cells were highly expressed with NOTCH3 and SMARCA4 or both NOTCH3 and SMARCA4 (Fig. 7J–L). These results suggest that the interaction of NOTCH3 and SMARCA4 plays a key role in the occurrence of MCA. The high expression of NOTCH3 and SMARCA4 indicates that the patients with colorectal adenocarcinoma are more likely to differentiate into mucinous adenocarcinoma.

**Table 2:** The relationship between NOTCH3, SMARCA4, MUC5AC and MUC2 from 3806 patients/3953 samples in 10 studies (cBioPortal for Cancer Genomics).

A	B	Neither	A not B	B not A	Both	Log_2_ odds ratio	*P*‐value	*q*‐Value	Tendency
NOTCH3	SMARCA4	3022	178	130	70	> 3	< 0.001	< 0.001	Co‐occurrence
NOTCH3	MUC2	1575	107	75	32	2.651	< 0.001	< 0.001	Co‐occurrence
NOTCH3	MUC5AC	1634	129	16	10	2.985	< 0.001	< 0.001	Co‐occurrence
SMARCA4	MUC2	1580	102	87	20	1.832	< 0.001	< 0.001	Co‐occurrence
MUC2	MUC5AC	1663	100	19	7	2.615	< 0.001	< 0.001	Co‐occurrence
SMARCA4	MUC5AC	1648	115	19	7	2.4	0.001	0.001	Co‐occurrence

## 4 Discussion

Colorectal cancer is a colorectal epithelial malignant tumor that includes colon cancer and rectal cancer and is one of the leading causes of cancer‐related mortality globally [53]. The pathological type of this cancer is mainly adenocarcinoma. With improvement in the living standards and changes in lifestyles, the rate of CRC incidence has risen, contributing to an ever‐increasing number of people in their twenties and thirties every year. Although the therapeutic effect of CRC patients has greatly improved, the overall prognosis of these patients remains poor. With the advancement in genomics and molecular pathology of cancer biomarkers, tumor treatment has become more individualized, requiring the refinement of cancer subtype classification according to its histological and genetic characteristics. MCA is a unique subtype with a high proportion in young patients with CRC. MCA sufferers' therapy and prognosis are dismal [3, 7, 8]. As a result, the current research into the molecular mechanisms underlying the incidence and progression of MCA is likely to give a more precise target for MCA diagnosis and therapy. It is thus of great significance to formulate a more reasonable and individualized treatment scheme for patients as well as to accelerate the speed of clinical diagnosis and prognosis toward improving the survival rate of patients.

Four different types of NOTCH receptors (NOTCH1–4) were observed in mammals, all of which were found to regulate cell homeostasis by participating in intercellular communication and regulating cell proliferation, differentiation, and apoptosis [9]. The carcinogenic and pathogenic functions of NOTCH signaling in human cancer were demonstrated for the first time in T‐cell acute lymphoblastic leukemia (T‐ALL), with the identification of chromosomal rearrangement involving the NOTCH1 locus [54]. The research reported > 50% of cases to have NOTCH1 gene mutations [55, 56]. In addition to NOTCH1, NOTCH3 signaling was also considered to be important in several types of cancers. NOTCH3 signaling plays a carcinogenic role in solid tumors, including CRC [57], breast cancer [58], and lung cancer [59]. It has been reported that the NOTCH3 levels were significantly upregulated in primary and metastatic CRC samples [21]. The nuclear NOTCH3 expression is associated with the recurrence of CRC [60]. In addition, DLL4 promotes the proliferation and differentiation of CRC cells by upregulating the expression of the NOTCH3 receptor in CRC [21]. However, the mechanism associated with the transcriptional regulation of NOTCH3 has not been reported so far, especially in terms of the role of NOTCH3 in the differentiation of CRC subtypes.

It has been confirmed that the activation of the NOTCH pathway requires a variety of transcriptional regulators [23], and the structural state of chromatin is extremely important for the activity of the transcriptional factors [24]. SMARCA4 controls transcription by modifying the chromatin structures and is involved in immunological response, inflammation, and embryonic development as the key member of the SWI/SNF chromatin remodeling complex [25, 26, 27]. There are currently only a few studies on the relationship between SMARCA4 and the NOTCH signaling pathway [23, 33, 34]. It was reported that SMARCA4 binds to SIRT1 and interferes with the deacetylation of p53, inhibiting cell proliferation, and inducing cell cycle arrest to promote the senescence of CRC cells by influencing the SMARCA4/SIRT1/p53/p21 signal axis [61]. It has also been reported that SMARCA4 regulates intestinal development through a NOTCH signal‐dependent mechanism in the duodenum [33]. Whether NOTCH3 regulates the occurrence and development of CRC and requires SMARCA4 remains unclear.

In this study, the cBioPortal for Cancer Genomics database analysis, TCGA database analysis, and IHC experiments revealed a strong correlation between NOTCH3 and SMARCA4 in CRC (Figs 1 and 2). Our results suggested that NOTCH3 could regulate the expression of SMARCA4 in CRC HT29 and HT115 cells (Fig. 2E–H). The involvement of NOTCH3 and SMARCA4 in the regulation of colon cancer was proved via direct interaction with endogenous and exogenous co‐IP and GST‐pulldown experiments (Fig. 3). The protein expression patterns of NOTCH3 and SMARCA4 may be closely related to the prognosis of patients with colon cancer (Fig. S2).

Previous studies on the NOTCH signaling pathway have demonstrated that NOTCH are essential for cell proliferation, invasion, metastasis, and apoptosis of CRC [14, 21, 62, 63, 64, 65, 66, 67]. However, different NOTCH members play distinct roles in the same tumor. Therefore, functional experiments, such as CCK‐8 proliferation assay, plate cloning assay, transwell assay, wound healing assay, and flow cytometry, revealed that NOTCH3 could interact with SMARCA4 directly in the growth, invasion and migration of CRC cells (Figs 4 and 5). EMT is an important biological process involved in the progression of cancer and refers to the transformation of epithelial cells from regular epithelial cells to irregular mesenchymal cells. The expressions of the mesenchymal markers N‐cadherin and vimentin increased, whereas that of the epithelial marker E‐cadherin decreased as a result of the normal physiological alterations associated with EMT [68]. The overexpression of NICD3 while knocking down SMARCA4 to detect the proliferation, invasion, and migration of CRC cells and the expression of the EMT markers (N‐cadherin, vimentin, and E‐cadherin) were studied (Fig. 5A–H, Fig. S3). Furthermore, the morphological changes provided a supporting evidence that the mesenchymal characteristics of CRC cells were significantly increased after NICD3 overexpression, and this phenomenon was markedly reversed after knocking down SMARCA4 (Fig. 5I). NOTCH3 was shown to be involved in the control of CRC progression, and it was revealed to depend on SMARCA4.

In addition, the possible targeting factors of NOTCH3 and SMARCA4 in colon cancer cells were screened through RNA‐seq, and, for the first time, we identified that MUC5AC and MUC2 may be the targeted factors for the co‐regulation of NOTCH3 and SMARCA4 interaction (Fig. 6A,B). NOTCH3 regulates the transcription and expression of MUC5AC and MUC2 in a SMARCA4‐dependent manner, and we also proved that NOTCH3 could regulate the promoter transcriptional activity of *MUC5AC* and *MUC2* by recruiting SMARCA4 (Fig. 6C–O). Further evidence suggests that NOTCH3 and SMARCA4 could regulate the progression of CRC cells by monitoring MUC5AC and MUC2 (Fig. S4). Early studies have demonstrated that the increased expression of MUC5AC and MUC2 proteins is one of the most obvious molecular features of MCA [3, 36, 37, 38, 39]. Moreover, the molecular profiles were found to be significantly different between MCA and non‐MCA, which indicated distinct carcinogenic mechanisms. MCA has also been linked to high‐frequency microsatellite instability (MSI‐H) in association with Lynch syndrome [69] and mutations in the Ras‐RAF‐MEK‐ERK pathway (RAS/MAPK pathway) [37]. Nevertheless, the elements that influence MCA differentiation and development and their impacts on patient prognosis remain unknown. Our results revealed that the interaction between NOTCH3 and SMARCA4 is the key regulatory factor in the differentiation of CRC subtypes.

Patients with MCA are now treated using the same conventional treatment recommendations as those with CRC. However, patients with mucinous adenocarcinoma require specific, customized treatment because of their poor response to standard chemotherapy and immunotherapy. Furthermore, multicolor fluorescence immunohistochemistry has revealed that the positive rate of cells co‐expressed with NOTCH3 and SMARCA4 was markedly higher in the MUC5AC and MUC2 co‐overexpression positive group and that there was a higher positive correlation between NOTCH3 and SMARCA4 in CRC patients with the co‐overexpression of MUC5AC and MUC2 (Fig. 7). These results further confirm the correlation between NOTCH3‐SMARCA4 and the differentiation of the CRC subtypes.

In‐depth investigation of NOTCH3 and SMARCA4's interaction mechanism and biological functions in transcriptional regulation and their participation in the regulation of downstream target factors MUC5AC and MUC2 are expected to help understand the molecular mechanisms involved in the differentiation and development of MCA. Meanwhile, relevant intervention treatment will be performed for NOTCH3 and its related targets, and relevant joint molecular diagnostic criteria will be formulated for the early clinical diagnosis of MCA. The establishment of staging evaluation and subtype refinement criteria will be able to provide a more accurate theoretical basis for interpreting the pathogenesis of MCA and improving the current prevention and treatment strategies of CRC, which has a broad clinical application prospect.

## 5 Conclusions

The present study revealed that SMARCA4 interacts with NICD3 directly to regulate the growth, invasion, and migration of CRC cells. In addition, NOTCH3 may target MUC5AC and MUC2 by recruiting SMARCA4, which has possible involvement in the differentiation and development of MCA. Our results may improve the understanding of the specific molecular mechanism of occurrence and development of MCA mediating by NOTCH3. We hope to provide a better basis for clinical patients with optimized individualized treatment, which is of great significance for improving the rate of clinical diagnosis, prognosis, and survival rate of MCA patients.

## Conflict of interest

The authors declare no conflict of interest.

## Author contributions

XY, YC, XZ and YH performed the experiment and drafted the manuscript. KX and JR conducted the clinicopathological data collection and analysis. YH, BW and YY analyzed the data. XY, YC, XZ, HH, WH and WW interpreted the results of the experiments. WW and WH conceived the research and finalized the manuscript. All authors have approved the final version of the manuscript and agree to be accountable for all aspects of the work.

### Peer review

The peer review history for this article is available at https://publons.com/publon/10.1002/1878‐0261.13296.

## Supporting information


**Fig. S1.** Screening of specific small‐interference RNA and overexpression adenovirus validation.
**Fig. S2.** The prognostic model analysis by the expression of NOTCH3 and SMARCA4 based on a CRC patient study cohort and the TCGA database.
**Fig. S3.** NOTCH3 regulates the progression of SW480 cells in a SMARCA4‐dependent manner.
**Fig. S4.** Changes in the migration and invasion abilities of HCT116 cells in different groups.
**Table S1.** Clinical characteristics of patients with CRC used for general IHC analysis.
**Table S2.** Clinical characteristics of patients with CRC used for multicolor Manual IHC analysis.
**Table S3.** Antibodies used in related experiments.
**Table S4.** The siRNA sequence used in the present study.
**Table S5.** Primer sequences used in qRT‐PCR.

## Data Availability

The datasets generated and/or analyzed during the current study are available from the corresponding author on reasonable request.
