# Supplementary material for: NICD3 regulates the expression of MUC5AC and MUC2 by recruiting SMARCA4 and is involved in the differentiation of mucinous colorectal adenocarcinoma
Source: Mol Oncol. 2022 Aug 11;16(19):3509–32. doi: 10.1002/1878-0261.13296 (PMC9533685; doi:10.1002/1878-0261.13296)
Supplement: Supplementary file 1 — Fig. S1. Screening of specific small‐interference RNA and overexpression adenovirus validation. Fig. S2. The prognostic model analysis by the expression of NOTCH3 and SMARCA4 based on a CRC patient study cohort and the TCGA database. Fig. S3. NOTCH3 regulates the progression of SW480 cells in a SMARCA4‐dependent manner. Fig. S4. Changes in the migration and invasion abilities of HCT116 cells in different groups. Table S1. Clinical characteristics of patients with CRC used for general IHC analysis. Table S2. Clinical characteristics of patients with CRC used for multicolor Manual IHC analysis. Table S3. Antibodies used in related experiments. Table S4. The siRNA sequence used in the present study. Table S5. Primer sequences used in qRT‐PCR. [file MOL2-16-3509-s001.docx]

**Supporting information**

**
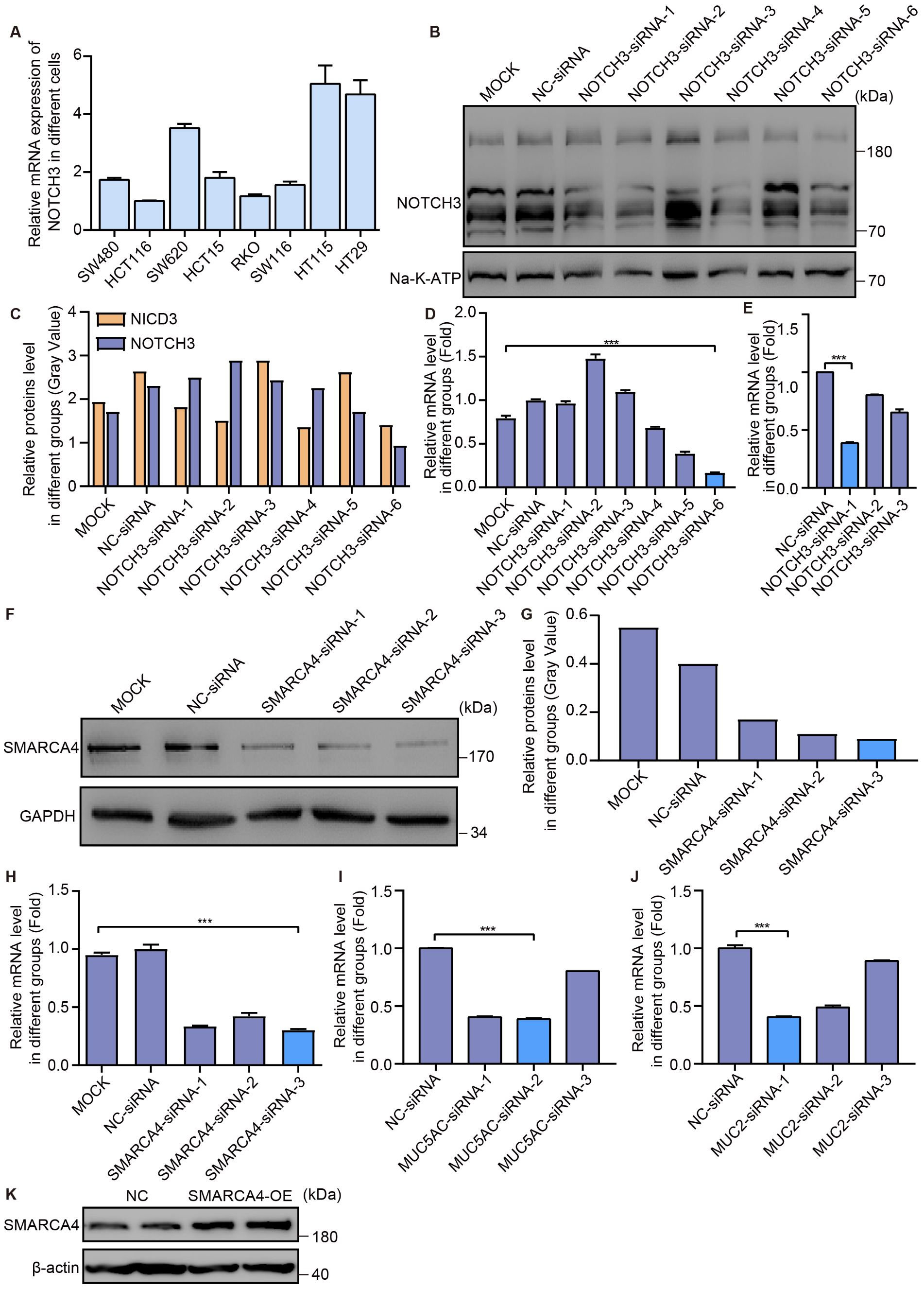
**

**Fig. S1. Screening of specific small-interference RNA and overexpression adenovirus validation.** (A) The relative mRNA expression of endogenous NOTCH3 in different colon cancer cells (*n* = 3). (B) WB detection diagram for specific siRNA screening of NOTCH3. (C) Statistical analyses of the relative gray value of WB detection in panel B. (D) qRT-PCR detection diagram for specific siRNA screening of NOTCH3 (*n* = 3). (E) qRT-PCR detection diagram for new specific siRNA screening of NOTCH3 (*n* = 3). (F) WB detection diagram for specific siRNA screening of SMARCA4. (G) Statistical analysis of the relative gray value of WB detection in panel F. (H) qRT-PCR detection diagram for specific siRNA screening of SMARCA4 (*n* = 3). (I) qRT-PCR detection diagram for specific siRNA screening of MUC5AC (*n* = 3). (J) qRT-PCR detection diagram for specific siRNA screening of MUC2 (*n* = 3). (K) The expression efficiency of SMARCA4 overexpression adenovirus detected by WB. Data are presented as mean ± SEM. Statistical analyses were performed using unpaired Student’s *t* tests. ^***^*P* < 0.001; NC, negative control; si, short interfering; OE, overexpression adenovirus; MOCK, HT29 cells.

**
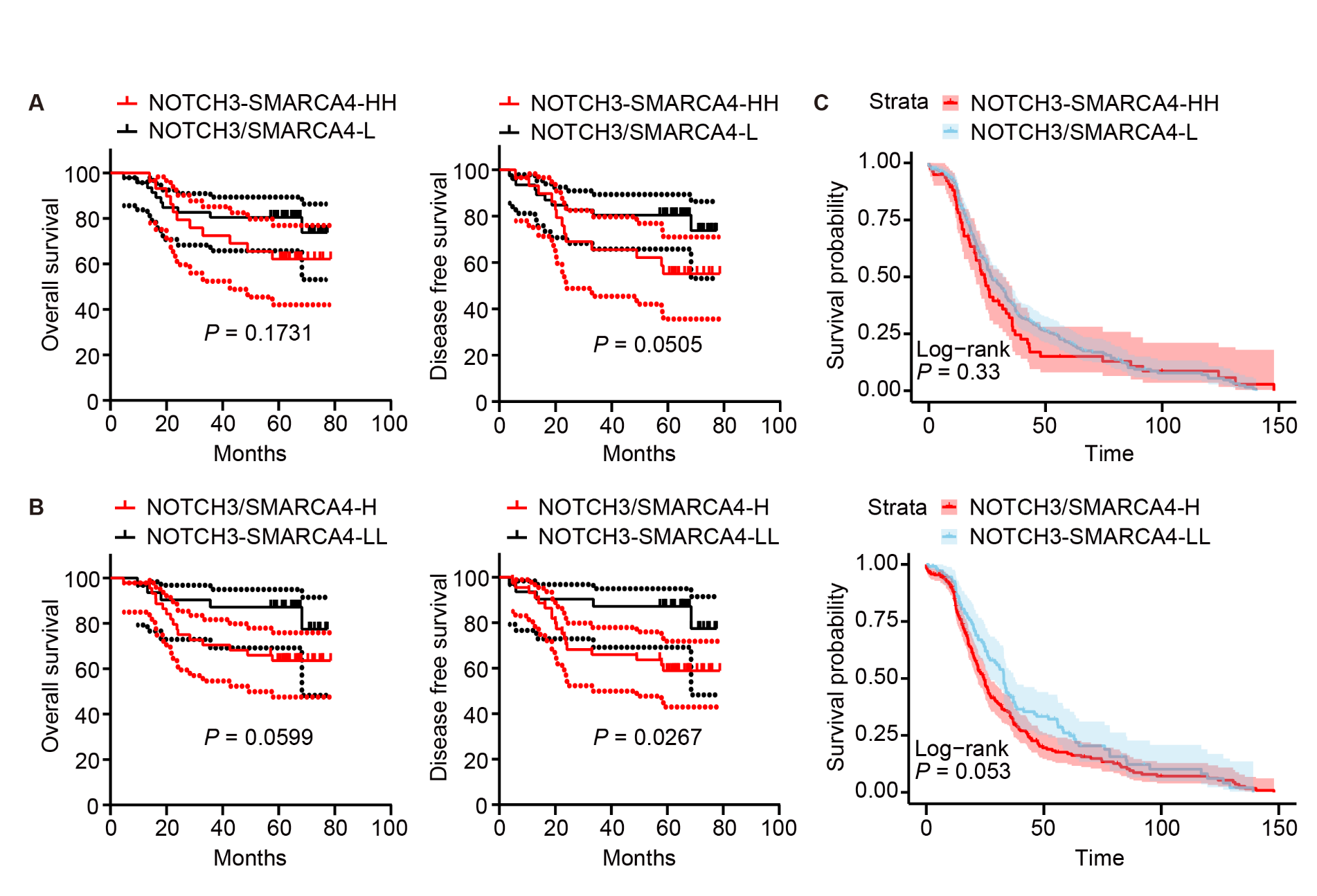
**

**Fig. S2. The prognostic model analysis by the expression of NOTCH3 and SMARCA4 based on a CRC patient study cohort and the TCGA database.** (A) The overall and disease-free survival analysis between patients with NOTCH3 and the SAMRCA4 simultaneous high-protein expression group and patients with NOTCH3 or SMARCA4 poor protein expression group (*n* = 75). (B) The overall and disease-free survival analysis between patients with NOTCH3 and/or SAMRCA4 high-protein expression group and patients with NOTCH3 and SMARCA4 poor protein expression group (*n* = 75). (C) Prognostic analysis of different expression patterns based on the TCGA database (*n* = 373). Log-rank (Mantel-Cox) test was used for the survival analysis. HH, represents a relatively high expression of both; LL, represents a relatively poor expression of both; H, represents a single relatively high expression; L, represents a single relatively poor expression.

**
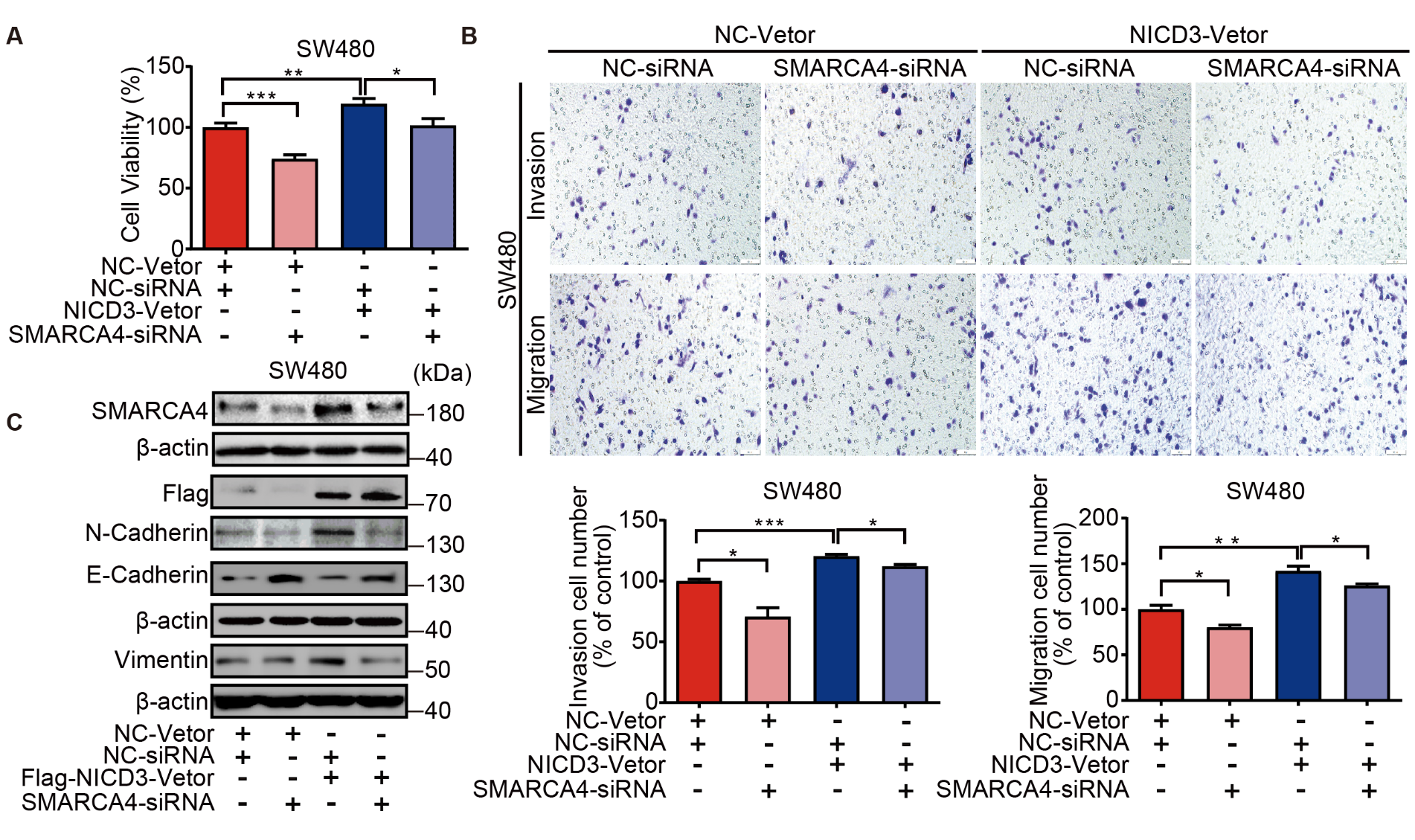
**

**Fig. S3. NOTCH3 regulates the progression of SW480 cells in a SMARCA4-dependent manner.** (A) CCK8 assay was performed to detect the proliferative activity of SW480 cells in different groups (*n* = 3). (B) Transwell invasion and migration assays were performed to detect the invasion and migration abilities of SW480 cells in different groups, and the related statistical analysis was performed (Scale bar: 50 μm

; *n* = 3). (C) The knockdown of SMARCA4 increased the E-cadherin expression and decreased the N-cadherin and vimentin expression in NICD3-overexpressed SW480 cells. Data are presented as mean ± SEM. Statistical analyses were performed using unpaired Student’s *t* tests. ^*^*P* < 0.05, ^**^*P* < 0.01, ^***^*P* < 0.001; NC, negative control; si, short interfering.

**
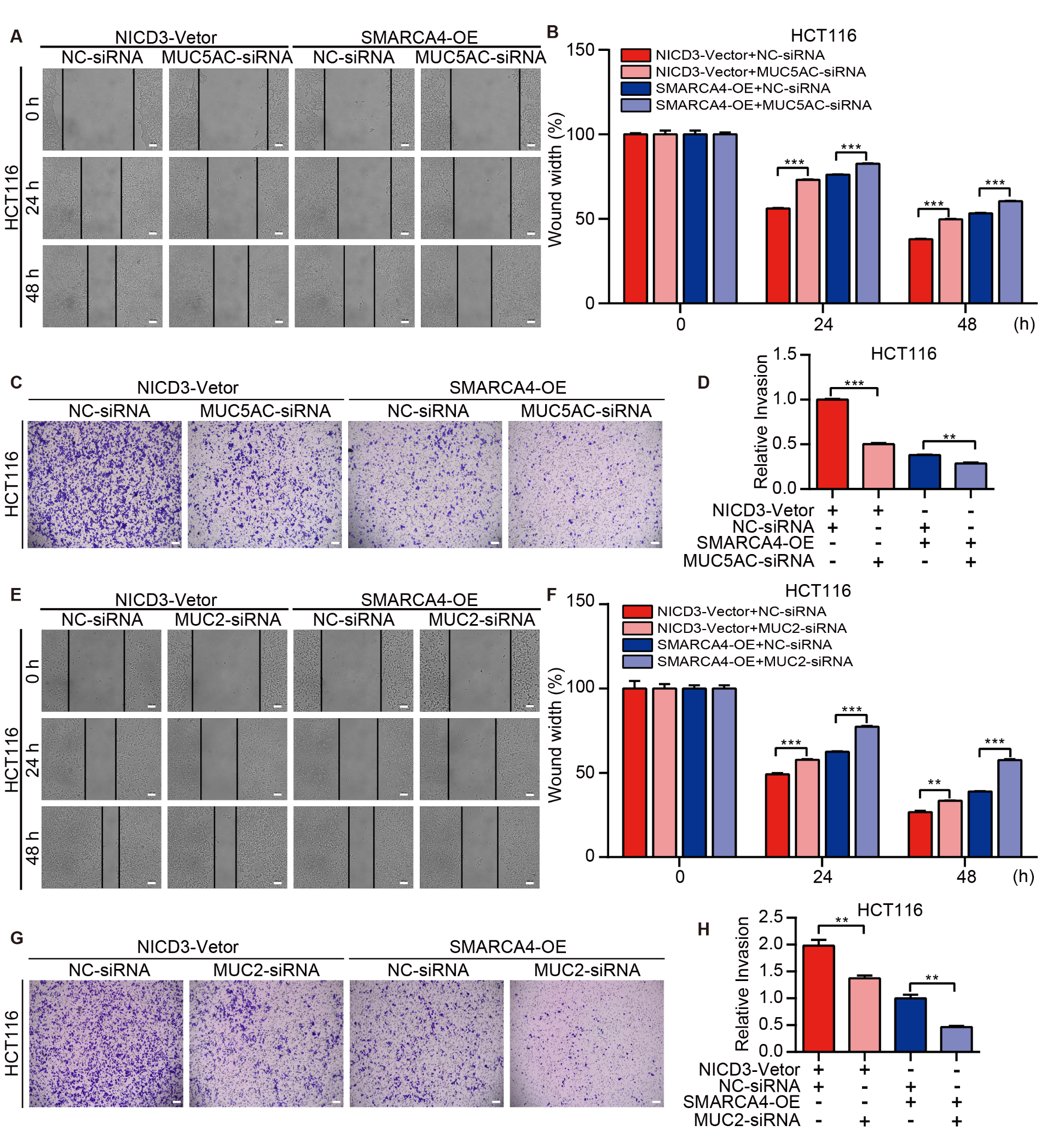
**

**Fig. S4.** **Changes in the migration and invasion abilities of HCT116 cells in different groups.** (A and B) Scratch assays were performed to detect the migration abilities of HCT116 cells with the overexpression of NICD3 or SMARCA4 simultaneously knocking down MUC5AC, and the related statistical analysis was performed (Scale bar: 100 μm; *n* = 3). (C and D) Transwell invasion assay was performed used to detect the invasion abilities of HCT116 cells with the overexpression of NICD3 or SMARCA4 simultaneously knocking down MUC5AC, and the related statistical analysis was performed (Scale bar: 400 μm; *n* = 3). (E and F) The scratch assays were performed to detect the migration abilities of HCT116 cells with the overexpression of NICD3 or SMARCA4 simultaneously knocking down MUC2, and the related statistical analysis was performed (Scale bar: 100 μm; *n* = 3). (G and H) Transwell invasion assay was performed to detect the invasion abilities of HCT116 cells with the overexpression of NICD3 or SMARCA4 simultaneously knocking down MUC2, and the related statistical analysis was performed (Scale bar: 400 μm; *n* = 3). Data are presented as mean ± SEM. Statistical analyses were performed using unpaired Student’s *t* tests. ^**^*P* < 0.01, ^***^*P* < 0.001; NC, negative control; si, short interfering; OE, overexpression adenovirus.

**Table S1.** Clinical characteristics of patients with CRC used for general IHC analysis.

| Characteristic | | | | No. of patients (n %) | |
| --- | --- | --- | --- | --- | --- |
| Number | | |  | 112（100%） |  |
|  |  |  |  |  |  |
| Age | | |  |  |  |
| Median (in years) | | |  | 55 |  |
| Range (in years) | | |  | 25-79 |  |
|  | | |  |  |  |
| Sex | | |  |  |  |
| Male | | |  | 61（54.5%） |  |
| Female | | |  | 51（45.5%） |  |
|  | | |  |  |  |
| Ethnicity | | |  |  |  |
| Asian | | |  | 112（100%） |  |
|  | | |  |  |  |
| T-Stage | | |  |  |  |
| T1 | | |  | 1 (0.9%) |  |
| T2 | | |  | 15 (13.4%) |  |
| T3 | | |  | 76（67.9%） |  |
| T4 | | |  | 20（17.8%） |  |
|  | | |  |  |  |
| N-Stage | | |  |  |  |
| N0 | | |  | 36（32.1%） |  |
| N1a | | |  | 11（9.8%） |  |
| N1b | | |  | 28（25%） |  |
| N2a | | |  | 17（15.2%） |  |
| N2b | | |  | 20（17.9%） |  |
|  | | |  |  |  |
| M-Stage | | |  |  |  |
| M0 | | |  | 87（77.7%） |  |
| M1 | | |  | 25（22.3%） |  |
|  | | |  |  |  |
| Pathological Stage | | |  |  |  |
| I | | |  | 14（12.5%） |  |
| II | | |  | 19（17.0%） |  |
| III | | |  | 54（48.2%） |  |
| IV | | |  | 25（22.3%） |  |

**Table S2.** Clinical characteristics of patients with CRC used for multicolour Manual IHC analysis.

| Characteristic | | | | No. of patients (n %) | |
| --- | --- | --- | --- | --- | --- |
| Number | | |  | 94（100%） |  |
|  |  |  |  |  |  |
| Age | | |  |  |  |
| Median (in years) | | |  | 65 |  |
| Range (in years) | | |  | 29-86 |  |
|  | | |  |  |  |
| Sex | | |  |  |  |
| Male | | |  | 46（48.9%） |  |
| Female | | |  | 48（51.1%） |  |
|  | | |  |  |  |
| Ethnicity | | |  |  |  |
| Asian | | |  | 94（100%） |  |
|  | | |  |  |  |
| T-Stage | | |  |  |  |
| T2 | | |  | 9 (9.6%) |  |
| T3 | | |  | 33（35.1%） |  |
| T4a | | |  | 42（44.7%） |  |
| T4b | | |  | 10（10.6%） |  |
|  | | |  |  |  |
| N-Stage | | |  |  |  |
| N0 | | |  | 58（61.7%） |  |
| N1 | | |  | 19（20.2%） |  |
| N2 | | |  | 17（18.1%） |  |
|  | | |  |  |  |
| M-Stage | | |  |  |  |
| M0 | | |  | 89（94.7%） |  |
| M1 | | |  | 5（5.3%） |  |
|  | | |  |  |  |
| Pathological Stage | | |  |  |  |
| II | | |  | 58（61.7%） |  |
| II-III | | |  | 29（30.9%） |  |
| III | | |  | 7（7.4%） |  |

**Table S3.** Antibodies used in related experiments.

| Reagent type  (species) or  resource | Designation | Source or reference | Identifiers | Additional information |
| --- | --- | --- | --- | --- |
| Antibody | Rabbit polyclonal to anti- NOTCH3 | Abcam | Cat. # ab23426 | (1:100) for Immunohistochemistry (IHC) |
| Antibody | Rabbit Polyclonal anti- SMARCA4/BRG1 | Proteintech | Cat. # 21634-1-AP | (1:1000) for WB  (1:200) for IHC |
| Antibody | Rabbit monoclonal anti- NOTCH3 | Cell signaling Technology | Cat. # 5276S | (1:1000) for western blot (WB)  (1:200) for immunoprecipitation (IP) |
| Antibody | Mouse monoclonal anti- SMARCA4 | Cell signaling Technology | Cat. #52251S | (1:1000) for western blot (WB)  (1:200) for immunoprecipitation (IP) |
| Antibody | Normal rabbit IgG | Proteintech | Cat. # B900610 | Dilute to the same concentration as the specific IP antibody |
| Antibody | Normal mouse IgG | Proteintech | Cat. # B900620 | Dilute to the same concentration as the specific IP antibody |
| Antibody | Rabbit monoclonal anti- MUC5AC | Cell signaling Technology | Cat. # 61193S | (1:400) for immunofluorescence (IF).  (1:150) for Immunohistochemistry (IHC) |
| Antibody | Mouse monoclonal anti- MUC2 | Abcam | Cat. # ab11197 | (1:500) for immunofluorescence (IF).  (1:300) for Immunohistochemistry (IHC) |
| Antibody | Rabbit Polyclonal anti- E-cadherin | Proteintech | Cat. # 20874-1-AP | (1:2000) for WB |
| Antibody | Rabbit Polyclonal anti- N-cadherin | Proteintech | Cat. # 22018-1-AP | (1:2000) for WB |
| Antibody | Rabbit Polyclonal anti- Vimentin | Proteintech | Cat. # 10366-1-AP | (1:1000) for WB |
| Antibody | Goat anti-Rabbit IgG (H+L) Cross-Adsorbed Secondary Antibody, Alexa Fluor 594 | ThermoFisher SCIENTIFIC | Cat. # A-11012 | (1:200) for IF |
| Antibody | Goat anti-Mouse IgG (H+L) Cross-Adsorbed Secondary Antibody, Alexa Fluor 488 | ThermoFisher SCIENTIFIC | Cat. # A-11001 | (1:300) for IF |
| HRP-linked secondary antiboay | Rabbit monoclonal antiboay IgG | Cell signaling Technology | Cat. # 7074S | (1:5000) for WB  (1:5000) for IHC |
| HRP-linked secondary antiboay | Mouse monoclonal antiboay IgG | Cell signaling Technology | Cat. # 7076S | (1:5000) for WB  (1:5000) for IHC |
| Antibody | Rabbit Polyclonal anti- GST | Proteintech | Cat. # 10000-0-AP | (1:1000) for WB |
| Antibody | Mouse monoclonal anti- His | Proteintech | Cat. # 66005-1-Ig | (1:5000) for WB |
| Antibody | Rabbit monoclonal anti-β-Actin | Cell signaling Technology | Cat. # 4970S | (1:1000) for WB |
| Antibody | DYKDDDDK Tag (D6W5B) Rabbit mAb | Cell signaling Technology | Cat. # 14793S | (1:1000) for WB  (1:100) for co-IP |
| Antibody | GFP tag Monoclonal antibody | Proteintech | Cat. # 66002-1-Ig | (1μg/mg protein) for co-  immunoprecipitation (co-IP)  (1:2000) for western blot (WB) |

**Table S4.** The siRNA sequence used in the present study.

| Primer Name | | Sequence(5’to3’) |
| --- | --- | --- |
| NOTCH3 siRNA-1 | Sense | GGUAGUAAUGCUGGAGAUUTT |
|  | Anti-sense | AAUCUCCAGCAUUACUACCTT |
| NOTCH3 siRNA-2 | Sense | GCCACAGACUGGAUGGACATT |
|  | Anti-sense | UGUCCAUCCAGUCUGUGGCTT |
| NOTCH3 siRNA-3 | Sense | CCAAGCGGCUAAAGGUAGATT |
|  | Anti-sense | UCUACCUUUAGCCGCUUGGTT |
| NOTCH3 siRNA-4 | Sense | UGGAUGAGUGCCUGAGCAATT |
|  | Anti-sense | UUGCUCAGGCACUCAUCCATT |
| NOTCH3 siRNA-5 | Sense | GGAUGAUUCAGAUGACACATT |
|  | Anti-sense | UGUGUCAUCUGCCUCAUCCTT |
| NOTCH3 siRNA-6 | Sense | CCAAUAAGGACAUGCAGGATT |
|  | Anti-sense | UCCUGCAUGUCCUUAUUGGTT |
| SMARCA4 siRNA-1 | Sense | CCAAGAAGAUGAAGAAGAUTT |
|  | Anti-sense | AUCUUCUUCAUCUUCUUGGTT |
| SMARCA4 siRNA-2 | Sense | CCGUCAAAGUGAAGAUCAATT |
|  | Anti-sense | UUGAUCUUCACUUUGACGGTT |
| SMARCA4 siRNA-3 | Sense | AGAAAGUGGCUCAGAAGAATT |
|  | Anti-sense | UUCUUCUGAGCCACUUUCUTT |
| Control-siRNA | Sense | UUCUCCGAACGUGUCACGUTT |
|  | Anti-sense | ACGUGACACGUUCGGAGAATT |
| NOTCH3-1- siRNA-1 | Sense | GAGAGCUGCAGUCAGAAUATT |
|  | Anti-sense | UAUUCUGACUGCAGCUCUCTT |
| NOTCH3-1- siRNA-2 | Sense | GAGGCAGACAUCAAUGAGUTT |
|  | Anti-sense | ACUCAUUGAUGUCUGCCUCTT |
| NOTCH3-1- siRNA-3 | Sense | GCGAUCAGGACAUCAAUGATT |
|  | Anti-sense | UCAUUGAUGUCCUGAUGGCTT |
| MUC2- siRNA-1 | Sense | GCGAGCAGUGUGUCUGUAATT |
|  | Anti-sense | UUACAGACACACUGCUCGCTT |
| MUC2- siRNA-2 | Sense | GCCCAUCUAUGAGGAGGAUTT |
|  | Anti-sense | AUCCUCCUCAUAGAUGGGCTT |
| MUC2- siRNA-3 | Sense | GCUGUACGUUGGAGUUCUATT |
|  | Anti-sense | UAGAACUCCAACGUACAGCTT |
| MUC5AC- siRNA-1 | Sense | GCCACUACAACCAGCACAATT |
|  | Anti-sense | UUGUGCUGGUUGUAGUGGCTT |
| MUC5AC- siRNA-2 | Sense | GCACAAGCCAUGUUUCCAUTT |
|  | Anti-sense | AUGGAAACAUGGCUUGUGCTT |
| MUC5AC- siRNA-3 | Sense | CCUACACACAGAACGACUUTT |
|  | Anti-sense | AAGUCGUUCUGUGUGUAGGTT |

**Table S5.** Primer sequences used in qRT-PCR.

| Primers | Sequence(5’to3’) |
| --- | --- |
| NOTCH3 - Forward | GCCACAGACTGGATGGACAC |
| NOTCH3 - Reverse | CGGATGTCAGCAGCAACCA |
| SMARCA4 - Forward | GCTCCGAGGTCTGATAGTGAA |
| SMARCA4 - Reverse | GCTGTCTGGATCTGGAATCTTC |
| MUC5AC - Forward | TGTTCTGCGACTACTACAAC |
| MUC5AC - Reverse | GCATCTTGTCCTCATCAAAG |
| MUC2- Forward | CGGTTCTCCAGTTTATTCCT |
| MUC2 - Reverse | TCACACTTCTTACAGCACTC |
| GAPDH - Forward | GTCTTCACCACCATGGAGAA |
| GAPDH - Reverse | TAAGCAGTTGGTGGTGCAG |
